# Computational Discovery of Intermolecular Singlet Fission Materials Using Many-Body Perturbation Theory

**DOI:** 10.1021/acs.jpcc.4c01340

**Published:** 2024-05-01

**Authors:** Xiaopeng Wang, Siyu Gao, Yiqun Luo, Xingyu Liu, Rithwik Tom, Kaiji Zhao, Vincent Chang, Noa Marom

**Affiliations:** † School of Foundational Education, University of Health and Rehabilitation Sciences, Qingdao 266113, China; ‡ Qingdao Institute for Theoretical and Computational Sciences, Institute of Frontier and Interdisciplinary Science, Shandong University, Qingdao, Shandong 266237, P. R. China; § Department of Materials Science and Engineering, Carnegie Mellon University, Pittsburgh, Pennsylvania 15213, United States; ∥ Department of Physics, Carnegie Mellon University, Pittsburgh, Pennsylvania 15213, United States; ⊥ Department of Chemistry, Carnegie Mellon University, Pittsburgh, Pennsylvania 15213, United States

## Abstract

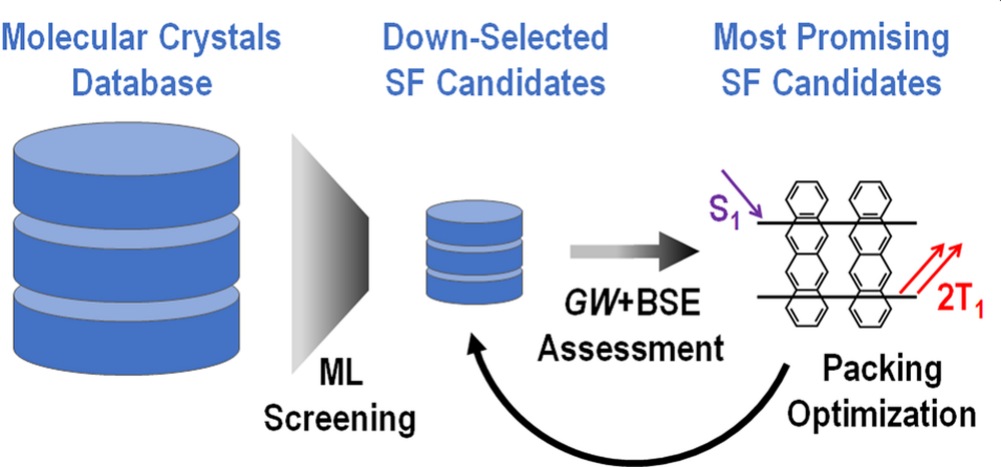

Intermolecular singlet fission (SF) is the conversion of a photogenerated singlet exciton into two triplet excitons residing on different molecules. SF has the potential to enhance the conversion efficiency of solar cells by harvesting two charge carriers from one high-energy photon, whose surplus energy would otherwise be lost to heat. The development of commercial SF-augmented modules is hindered by the limited selection of molecular crystals that exhibit intermolecular SF in the solid state. Computational exploration may accelerate the discovery of new SF materials. The GW approximation and Bethe–Salpeter equation (GW+BSE) within the framework of many-body perturbation theory is the current state-of-the-art method for calculating the excited-state properties of molecular crystals with periodic boundary conditions. In this Review, we discuss the usage of GW+BSE to assess candidate SF materials as well as its combination with low-cost physical or machine learned models in materials discovery workflows. We demonstrate three successful strategies for the discovery of new SF materials: (i) functionalization of known materials to tune their properties, (ii) finding potential polymorphs with improved crystal packing, and (iii) exploring new classes of materials. In addition, three new candidate SF materials are proposed here, which have not been published previously.

## Introduction

### Motivation and Prospective Applications

**Figure 1:**
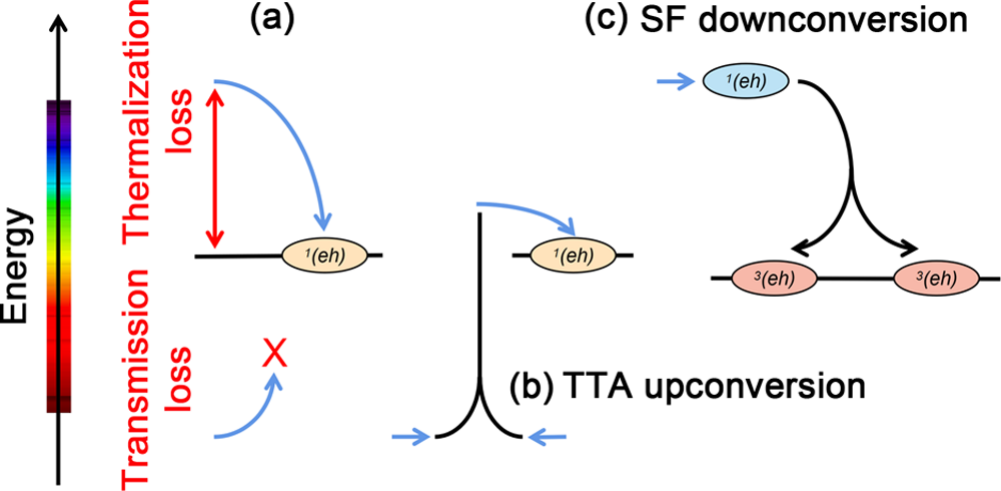
(a) Spectral losses of solar cells. (b) TTA upconversion. (c) SF downconversion. The blue arrows indicate photons. The horizontal black lines and ovals indicate excited states and excitons.

The light-to-electrical power conversion efficiency of solar energy is constrained by limitations at both the high end and the low end of the photon energy spectrum,^1−7^ illustrated in Figure 1a.

At the high-energy end of the solar spectrum, the efficiency is constrained by the Shockley–Queisser (SQ) limit.^8^ In a conventional single-junction solar cell, each absorbed photon is converted into one charge carrier. Therefore, a high-energy photon can generate only one photoexcited electron–hole pair, with the surplus energy above the absorber's bandgap, *E*_g_, lost to heat. This is known as the thermalization loss. At the low-energy end of the solar spectrum, photons with energy below the band gap of the absorber cannot be harvested. This is known as the transmission loss. Taking into consideration the thermalization and transmission losses, the theoretical efficiency limit is around 30% for a band gap of 1.1 eV.^8,9^ Hence, silicon, with a band gap of 1.1 eV, stands out as a particularly efficient material. We note that the Shockley–Queisser (SQ) limit is an upper bound on (single-junction) solar cell efficiency because there are other loss mechanisms, such as transport losses.^1,10−15^

In so-called third-generation solar cells,^16−18^ exciton upconversion and downconversion pathways are exploited to overcome the thermalization and transmission losses, as shown in Figure 1. The loss of sub-band-gap photons may be mitigated by optical upconversion of two low-energy photons into one high-energy photon, which is subsequently absorbed by the solar cell. In organic materials, upconversion can be achieved through triplet–triplet annihilation (TTA),^19−27^ where two low-energy triplet excitons are converted into one higher energy singlet exciton. Singlet fission (SF),^28^ the reverse process of TTA, is a form of multiple exciton generation wherein a high-energy photogenerated singlet exciton splits into two triplet excitons,^29^ which are subsequently converted into charge carriers. Thus, the excess photon energy is converted into an extra charge carrier instead of being lost to heat.

The occurrence of SF was first proposed in order to explain the delayed fluorescence in anthracene crystals in 1965.^30^ Later, SF was also observed in tetracene,^31,32^ perylene,^33^ and photosynthetic organisms.^34−36^ In 1979, Dexter suggested that SF could be used to improve the performance of photovoltaic cells.^37^ However, SF languished in obscurity for decades until in 2006 Hanna and Nozik suggested that using SF materials together with conventional absorbers could increase the conversion efficiency of solar cells to 42%,^38^ surpassing the SQ limit. SF, in and of itself, does not improve the performance of solar cells because although the number of excitons is doubled, i.e., a quantum yield of 200%, their energy is approximately halved. Rather, SF materials are employed to augment the conventional absorber in a multijunction configuration, such that the SF material absorbs the high-energy photons, reducing thermalization losses, and the conventional absorber absorbs the remaining photons.^38,39^

Several architectures have been proposed for SF augmented solar cells, in which the SF materials are interfaced with different materials.^39^ In organic photovoltaic (OPV) devices,^40^ the SF material is paired with an organic absorber. A representative example is a pentacene/C_60_ junction, where downconverted triplet excitons in pentacene dissociate at the interface into electrons in the C_60_ acceptor and holes in the pentacene donor.^41−47^ A challenge of this architecture is the trade-off between the diffusion of triplet excitons to the interface, which increases with decreasing thickness of the SF material, vs the photon absorption and subsequent SF yield, which increases with increasing thickness.^43,46,48,49^ In hybrid organic/inorganic quantum dot (QD) devices, the SF material is paired with an inorganic QD, whose energy levels can be precisely tuned to match up with those of the SF material.^50−52^ Dye-sensitized solar cells (DSSCs) consist of a layer of SF dye molecules adsorbed on a porous oxide semiconductor surface, such that triplet exciton loss is reduced thanks to a short diffusion distance.^53−57^ Morphology control has proven challenging in this type of devices.^39,53,58^ In perovskite-based cells the SF material is paired with a hybrid organic–inorganic halide perovskite absorber.^59,60^ However, the relatively large band gap of these absorbers makes it difficult to pair them with SF materials with matching triplet energies.^61^ Finally, silicon-based solar cells can be augmented with SF materials.^62−66^ This is arguably the most promising avenue thanks to the widespread commercial utilization of silicon-based cells. Other commonly used inorganic absorbers, such as GaAs, may also be augmented with SF materials.^39,67,68^

### Requirements for Efficient Singlet Fission

Spin conservation requires the involvement of two molecules or two connected molecular subunits in intermolecular or intramolecular SF, respectively:
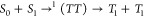
1where *S*_0_ is a chromophore in its ground state, *S*_1_ a chromophore in its lowest singlet excited state, ^1^(*TT*) a correlated triplet pair^69−71^ on two chromophores with a total spin multiplicity of 1, and *T*_1_ a chromophore in its lowest triplet excited state. The primary requirement of SF materials is that the SF process should be thermodynamically favorable.^28^ For SF to be exothermic, the *S*_1_ energy should be larger than twice the *T*_1_ energy:
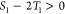
2We note that the energy of the bound triplet pair in eq 1, ^1^(*TT*), may be slightly smaller than 2*T*_1_. A positive energy driving force, *S*_1_ – 2*T*_1_, is preferred in practical applications because it is associated with a fast SF rate. A positive *S*_1_ – 2*T*_1_ may also suppress the competing reverse process of TTA. However, an overly large *S*_1_ – 2*T*_1_ leads to an energy loss and reduces the conversion efficiency. In addition, a large *S*_1_ energy may result in a too high absorption threshold and a small *T*_1_ energy may cause difficulties in triplet harvesting. Thus, it is considered optimal for a material to have an *S*_1_ energy that is slightly larger than 2*T*_1_. In some SF materials, such as crystalline tetracene, SF is slightly endothermic^72,73^ and driven by an entropy gain.^74,75^ In such materials the triplet yield may still be sufficiently high if SF is much faster than any competing radiative and nonradiative decay processes.

In addition to the thermodynamic driving force for SF, ideal SF materials need to meet the following requirements in order to facilitate the steps of absorption, SF, diffusion, and harvesting in a solar cell with minimal losses:^28,39,76^

1. Strong absorption in the energy range around *S*_1_ in order to absorb as many photons as possible with minimal film thickness.
2. High SF rate: SF must be much faster than all competing processes, especially its reverse process, TTA, and the typical loss mechanism of singlet energy transfer to other layers (for example, from the pentacene layer to the C60 acceptor layer in pentacene/C60 OPV devices). Other singlet exciton loss mechanisms are fluorescence and intersystem crossing (ISC) from *S*_1_ to *T*_1_. The spin conserving conversion from *S*_1_ to ^1^(*TT*) is usually ultrafast, ocurring within a picosecond or less.^77^ A fast dissociation of the correlated triplet pair ^1^(*TT*) into independent triplet excitons is additionally desired.
3. A long triplet lifetime is necessary for the diffusion of generated triplet excitons to the interface. Ideally, triplet harvesting should be faster than loss mechanisms such as TTA, phosphorescence, nonradiative quenching, static trapping, and interactions with charges.^78−81^ A high triplet exciton diffusion rate is also desirable. The orientation, crystallinity, and packing of molecules play a large role in triplet quenching^82,83^ and triplet exciton diffusivity.^84^ Singlet excitons typically hop from molecule to molecule by Förster resonance energy transfer (FRET) mediated by dipole–dipole interactions, a relatively long-range interaction that can lead to fast exciton motion.^85^ Triplet excitons can only transfer their energy through exchange coupling, known as Dexter energy transfer, because the triplet-excited-state to singlet-ground-state transition is a spin-forbidden process.^86^ Dexter energy transfer relies on wave function overlap between neighboring molecules, rendering it a much shorter-range process compared to FRET.^81^ The first requirement of high absorptivity also reduces the required thickness of the SF layer, i.e., the diffusion distance of triplet excitons. We note that although competing TTA is generally regarded as a loss mechanism, in the special case of tetrtacne, in which SF is nearly isoergic, singlet and triplet excitons can interconvert via SF and TTA and their transport is strongly coupled, leading to enhancement in the effective triplet exciton diffusion constant.^87^
4. In order to harvest the triplet excitons, the *T*_1_ energy of the SF material should be slightly higher than the conduction band edge of the material it is interfaced with for instance, the 1.1 eV of silicon. Any surplus *T*_1_ energy above this is lost.
5. Stability: In order for SF-augmented devices to be commercially viable, the SF material should have a long lifetime. This means that the chromophores themselves, as well as their crystal structure, should be thermally and chemically stable under operating conditions, upon exposure to light, heat, and possibly air.

### Intermolecular Singlet Fission Materials

Presently known SF materials do not meet all of the aforementioned requirements. Although there are hundreds of SF chromophores, they mainly belong to restricted chemical families including acenes, rylenes, benzofuran derivatives, diketopyrrolopyrroles, and carotenoids.^28,39,88^ Acenes stand out as the most extensively studied SF materials. Although unsubstituted acenes are not promising for commercial applications due to their instability and poor solubility,^89^ they have served as benchmark systems for studying SF mechanisms, developing new SF materials, and optimizing device architectures. Within the acene family, the SF driving force, *S*_1_ – 2*T*_1_, increases with the backbone length.^73,90^ SF in crystalline pentacene is slightly exoergic, whereas SF in tetracene is slightly endoergic.^72,73,91^ In hexacene, the overly high driving force leads to a large energy loss.^92^ Anthracene and its derivatives are more likely to undergo TTA than SF, owing to too negative *S*_1_ – 2*T*_1_.^26^ The exothermicity and endothermicity of SF in pentacene and tetracene, respectively, lead to opposite performance advantages and disadvantages. The energetically favorable SF in pentacene results in a large quantum yield and a fast SF rate. However, pentacene’s *T*_1_ energy of 0.86 eV^72^ is too low to be compatible with silicon. In comparison, the *T*_1_ energy of tetracene is 1.25 eV,^72^ making it compatible with more photovoltaic materials, including silicon. However, SF in tetracene is endoergic by about 0.21 eV,^72^ and driven by entropy gain, which leads to a slower rate than in pentacene.^74,75^ As a result, a thicker layer of tetracene than pentacene is required in order to achieve peak internal quantum efficiency in devices,^43,46,48,49^ but triplet diffusion losses become more significant for thicker layers. Another prominent acene derivative is rubrene, in which SF and TTA may occur simultaneously,^93−96^ precluding its application with high efficiency.

Compared to acenes, rylenes are more stable and exhibit stronger absorption. Unsubstituted oligorylenes have been computationally predicted to be SF candidates.^97−100^ SF has been experimentally observed in perylene single crystals^101,102^ and rylene derivatives,^88^ such as perylene diimide (PDI),^103^ PDI derivatives,^103−105^ and *tert*-butyl-substituted terrylene.^106^ The SF in perylene crystals is ultrafast (≪50 fs), but with a low triplet yield of 50%.^102^ In PDI, SF is endothermic by 0.2–0.3 eV^103,104^ and driven by entropy gain similar to tetracene. Although the triplet yield in PDI films is 140%, the rate constant is as slow as 180–3800 ps.^104,107,108^ SF in *tert*-butyl substituted terrylene has been reported to be endothermic by only 70 meV with a higher triplet yield of close to 200% within 320 ps.^106^ For the benzofuran derivative, 1,3-diphenylisobenzofuran (DPIBF), a fast SF rate of about 15 ps has been reported.^109^ The triplet energy of DPIBF is 1.42 eV,^110^ closely matching the 1.4 eV band gap of GaAs. The triplet yield of DPIBF is sensitive to its crystal structure.^54,55^ The triplet yield in the polymorph that undergoes efficient SF was reported to be 200% at 77 K,^109^ but 140% at room temperature.^55^ In addition, DPIDF is not sufficiently stable to be viable in device applications. For further discussion of known SF materials we refer the reader to refs (28, 88, and 111).

### Scope of This Review

Considering the limitations of known SF materials, new materials are desired for improving SF performance in practical applications. Computer simulations can help explore the chemical space and guide synthesis and characterization efforts in promising directions. In this Review, we focus on computational discovery of crystalline intermolecular singlet fission materials. Crystalline materials have the advantages of uniform, reproducible electronic and optical properties, as well as better transport,^112^ thanks to their ordered structure. For example, in ref (80) the performance of crystalline and amorphous 6,13-bis(triisopropylsilylethynyl)pentacene (TIPS-pentacene) was compared. The initial step of singlet fission was found to be similarly efficient in both phases. However, in the amorphous material appreciable losses were apparent prior to triplet pair separation. Hence, it was concluded that solution-processable, crystalline material should be targeted for optimal triplet yields in singlet fission.

The GW approximation and Bethe–Salpeter equation (GW+BSE), derived from many-body perturbation theory, is currently the state-of-the-art method for calculating the excited-state properties of molecular crystals with periodic boundary conditions. In the following, we briefly review the GW+BSE method. We proceed to discuss excitons in molecular crystals and demonstrate the use of GW+BSE simulations to assess candidate materials for SF in the solid state. Finally, we present examples for how GW+BSE can be integrated into materials discovery workflows and combined with low-cost models (physical or machine learned) to perform preliminary large-scale screening. The applications presented here focus mainly on our group’s work.^26,99,100,113−119^ Aggregated analyses of all the candidate SF materials found in our previous studies are presented. New results are presented for the performance of the ML models from ref (117) for additional materials, and three new SF candidates are proposed here, which have not been published previously. Other aspects of SF are discussed elsewhere.^28^ Additional reviews have focused on SF mechanisms,^77,120−123^ SF chromophores,^28,77,88,111^ SF photovoltaic devices,^39,84,88,122,124,125^ theoretical and computational modelings,^77,123,126^ spectroscopic techniques,^127,128^ and triplet pair states.^70,129,130^

## Brief Review of GW+BSE Methodology

### Formalism

Computational discovery and design of SF materials (as well as TTA and thermally activated delayed fluorescence (TADF) chromophores) requires evaluating singlet and triplet excitation energies. Time-dependent density functional theory (TDDFT)^131^ is often used to evaluate the excitation energies of isolated molecules. TDDFT is formally exact, but in practice its performance depends to a great extent on the choice of approximation for the exchange-correlation (xc) functional.^132−135^ TDDFT offers an appealing balance between accuracy and efficiency, if a judicious choice of approximation for the exchange-correlation functional is used.^133,134^ Hence, TDDFT has been widely used to search for SF,^136−139^ TTA,^27,140,141^ and TADF chromophores.^132,142,143^ However, because TDDFT does not easily lend itself to periodic implementations, for the most part its use is limited to isolated molecules and clusters. To some extent, it may be possible to mimic some of the effects of periodicity by conducting calculations for dimers or embedded clusters of molecules.^123,144−146^ Although these approaches may be able to capture the effect of the local environment of a molecule in a crystal by explicitly considering its nearest neighbors, and to approximate the presence of the surrounding medium by embedding, they cannot fully reproduce the evolution of molecular orbital energies into a band structure and the delocalization of excitons in molecular crystals (discussed in more detail below).^71,73,91,98,116,147−150^

**Figure 2:**
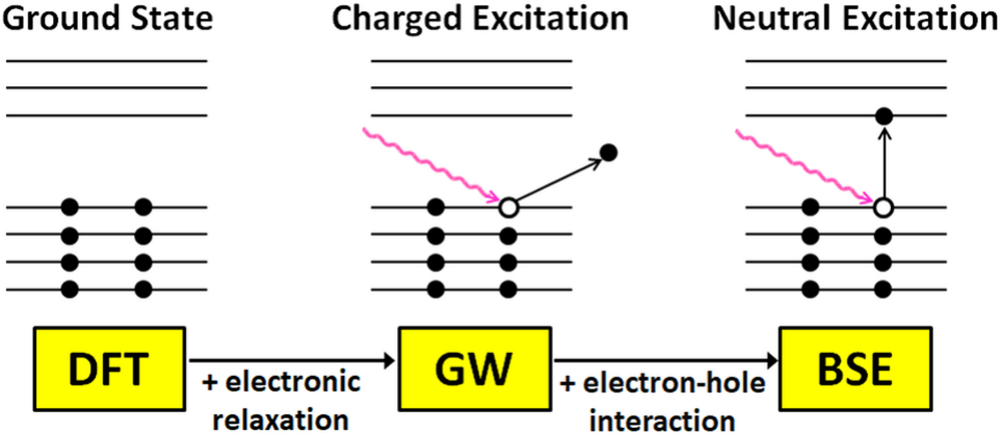
Illustration of the three-step workflow of GW+BSE. Full circles represent electrons, empty circles represent holes, and wiggly lines represent photons.

The excited-state properties of molecular crystals may be calculated with periodic boundary conditions using many-body perturbation theory (MBPT) within the GW approximation, where G stands for the one particle Green’s function and W stands for the screened Coulomb interaction,^151−153^ and the Bethe–Salpeter equation (BSE).^151−155^ GW+BSE calculations are a three-step process, as illustrated in Figure 2. The first step is obtaining approximate ground state wave functions and eigenvalues from a mean-field theory, typically density functional theory (DFT). The second step is performing the GW calculation. Typically, this is performed non-self-consistently (G_0_W_0_), owing to considerations of computational cost. The GW formalism provides a description of charged excitations, where an electron is added or removed from the system. GW accounts for the renormalization of the ground-state energy spectrum due to the polarization response of the system to the charge created by electron addition or removal. These updated energies are known as quasi-particle energies (if a self-consistent form of GW is used then the wave functions are also updated). The information provided by GW corresponds to quantities that can be measured in, e.g., photoemission spectroscopy (PES) experiments. It includes the quasi-particle band structure, the band edge positions, and the fundamental band gap. In the third step the GW quasi-particle energies are fed into a Bethe–Salpeter equation (BSE),^155^ which is solved to produce electron–hole excitation energies, i.e., neutral excitations. The BSE step provides information on optical properties, including singlet and triplet excitation energies, the optical gap (which differs from the fundamental gap by the exciton binding energy), the optical absorption spectrum, and exciton wave functions. The following is a brief description of the GW+BSE formalism. For in-depth reviews, the reader is referred to refs (147 and 156−164).

The DFT eigenvalues and eigenfunctions that serve as the starting point for GW+BSE are obtained by self-consistently solving the Kohn–Sham (KS) equation:^165^
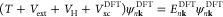
3where  are the KS eigenvalues and  are the KS eigenfunctions. The KS Hamiltonian comprises the kinetic energy, *T*, the external potential of the nuclei, *V*_ext_, the Hartree potential arising from the electrostatic interactions between electrons, *V*_H_, and an approximate functional for the exchange-correlation potential, . The KS equation maps the fully interacting many-electron system onto an effective one-electron system with the same ground-state density, given by
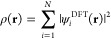
4where *N* is the number of electrons. In each iteration a new set of KS eigenvalues and eigenfunctions is obtained, and the electron density and KS Hamiltonian are updated until convergence is achieved.

Next, in the GW step, the KS eigenvalues and eigenfunctions,  and , serve as the initial guess. The quasi-particle eigenvalues and eigenvectors,  and , are then obtained by solving the following Dyson equation, which formally resembles the Kohn–Sham equation, but with the exchange-correlation functional replaced by the self-energy operator, Σ, which is nonlocal, energy-dependent, and non-Hermitian:^151^
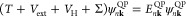
5Within the non-self-consistent *G*_0_*W*_0_ approach, the quasi-particle excitation energies, , are obtained from one-shot first-order perturbative corrections to the mean-field eigenvalues, , by solving
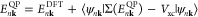
6G_0_W_0_ calculations employ the diagonal approximation, whereby off-diagonal elements of the self-energy are neglected, based on the assumption that the mean-field orbitals are sufficiently similar to the GW Dyson orbitals.

Finally, neutral excitation energies and the corresponding exciton wave functions (EWF) are calculated by solving the BSE for each exciton state, *S*:
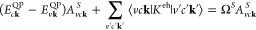
7where  and  are the GW quasi-particle eigenvalues of the conduction (c) and valence (v) states involved in a direct transition at *k*-point, *k*,  is the exciton wave function in the quasi-particle state representation, Ω^*S*^ is the excitation energy, and *K*^eh^ is the electron–hole interaction kernel. Within the Tamm–Dancoff approximation only transitions from valence states to conduction states are considered, i.e., the off-diagonal blocks of the electron–hole interaction kernel are set to zero.^166,167^ The screened Coulomb potential matrix elements contained in the BSE kernel ensure that long-range (nonlocal) electron–hole interactions are properly described.^162^ This contributes to the success of the BSE formalism in treating charge transfer (CT) excitations.^168−171^ In comparison, TDDFT based on standard (semi)local exchange-correlation functionals underestimates the energies of CT excited states due to the lack of long-range electron–hole interaction. This can be corrected by mixing a range-dependent fraction of exact (Fock) exchange with semilocal exchange and correlation in long-range corrected range-separated hybrid functionals.^132,143,172^ The BSE exciton wave function (EWF) may be visualized in real space or reciprocal space.^173,174^ In real space, the EWF can be expressed as
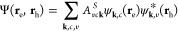
8where **r**_e_ and **r**_h_ are the real space positions of the electron and hole for exciton state *S*. For visualization purposes, the hole position, **r**_h_, may be fixed, such that the two-particle exciton wave function in eq 8 becomes an electron charge density with only one electron position variable:
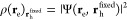
9 can then be plotted with respect to the chosen hole position. To calculate absorption spectra, the imaginary part of the dielectric function can be expressed as
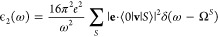
10where  and **v** is the velocity operator along the direction of the polarization of light, **e**.

### Limitations

Several benchmark studies have assessed the accuracy of GW and GW+BSE for isolated molecules, for which high-level quantum chemistry reference values can be obtained.^175−189^ Similar benchmarks for molecular crystals may be possible when periodic implementations of quantum chemistry methods^190,191^ become sufficiently mature to handle systems of this size. The accuracy of GW+BSE is limited by errors incurred as a result of the approximations introduced at each of the three steps. At the DFT level, local and semilocal exchange-correlation functionals suffer from the self-interaction error (SIE), a spurious repulsion of an electron from its own density.^192^ Some of the manifestations of the SIE are band-gap underestimation and destabilization of highly localized orbitals.^178,181^ In some cases, errors stemming from the exchange-correlation functional are not fully corrected by non-self-consistent G_0_W_0_,^178,181^ which may lead to a persistent underestimation of the quasi-particle band gap. Additional errors may stem from the GW approximation itself because higher-order terms in the expansion of the self-energy (contained in the vertex) are neglected^193−196^ and from the diagonal approximation employed in G_0_W_0_, which prevents intermixing of mean-field states.^179,182,197^ Additional numerical errors may arise from the need to carefully converge expressions involving sums over states with respect to the number of empty bands^113,198^ and the handling of the frequency dependence of the self-energy, for which some methods, such as the Hybertsen–Louie generalized plasmon-pole approximation, have been shown to yield significant errors in some cases.^167,199^

Errors in the GW quasi-particle energies propagate to the BSE calculation. Further errors are caused by the approximations employed in the BSE step. The BSE optical spectrum is sensitive to numerical settings, such as *k*-point sampling, which must be carefully converged.^113,116^ The Tamm–Dancoff approximation introduces errors, which have been shown to be more significant for some materials than others. It has been suggested that these errors are smaller for systems with extended excitons than for systems with localized excitons.^200−203^ For example, for oligoacene molecular crystals, it has been shown that the Tamm–Dancoff approximation results in negligible changes within 0.1 eV in the lowest excitation energies.^73,147,204^ For isolated molecules, the Tamm–Dancoff approximation has been shown to fortuitously improve the agreement of both triplet and singlet excitation energies obtained from GW+BSE with reference values obtained from higher level theories. It has been argued that this is because the Tamm–Dancoff approximation mitigates triplet instabilities in the BSE approach.^183,205^ Another approximation used in most BSE implementations is neglecting the dynamical screening and instead using a frequency-independent approximation for the dielectric screening by taking the zero-frequency static limit of the screened Coulomb interaction, *W*. This can also introduce errors for some materials.^206^ In addition, neglecting indirect (phonon-assisted) transitions may introduce errors in the BSE absorption spectrum of materials with an indirect band gap.^207^ Standard GW+BSE implementations fail to describe states with double-excitation character,^170,208,209^ although very recent developments show promise in that respect.^210^ The standard GW+BSE implementation also cannot describe the correlated biexciton state that may be relevant for SF.^71^

Despite its aforementioned limitations, GW+BSE is currently the state-of-the-art method for calculating the excited-state properties of periodic systems in general and molecular crystals in particular. All the results presented below were obtained with the BerkeleyGW^167^ implementation of GW+BSE, using the Perdew–Burke–Ernzerhof (PBE)^211^ DFT functional as a starting point. Further computational details are provided in refs (99, 100, 113−116, 118, and 119).

## Results and Discussion

### Excitons in Molecular Crystals

Unlike the excited states of isolated molecules, excitons in molecular crystals may exhibit a momentum dependence in the form of dispersed bands, similar to the electronic band structure.^71,203^ The EWFs of molecular crystals differ from those of isolated molecules in their spatial distribution. For an isolated molecule, the electron and hole may be located on the same part of the molecule or on different parts of the molecule, for example, intramolecular CT excited states in donor–acceptor complexes.^132^ In molecular crystals, excitons are often classified as having either a Frenkel character or a charge transfer character. In a Frenkel exciton the electron and hole are localized on the same molecule. In an exciton with an intermolecular CT character the electron and hole are localized on different molecules. The exciton character may be affected by, e*.*g*.*, the molecule size and the repulsive contribution stemming from the electron–hole exchange interaction.^171,212^ Excitons in real materials are often not purely Frenkel or CT excitons but rather have a mix of Frenkel and intermolecular CT character, with some probability of finding the electron on the same molecule as the hole and some probability of finding the electron on neighboring molecules.

**Figure 3:**
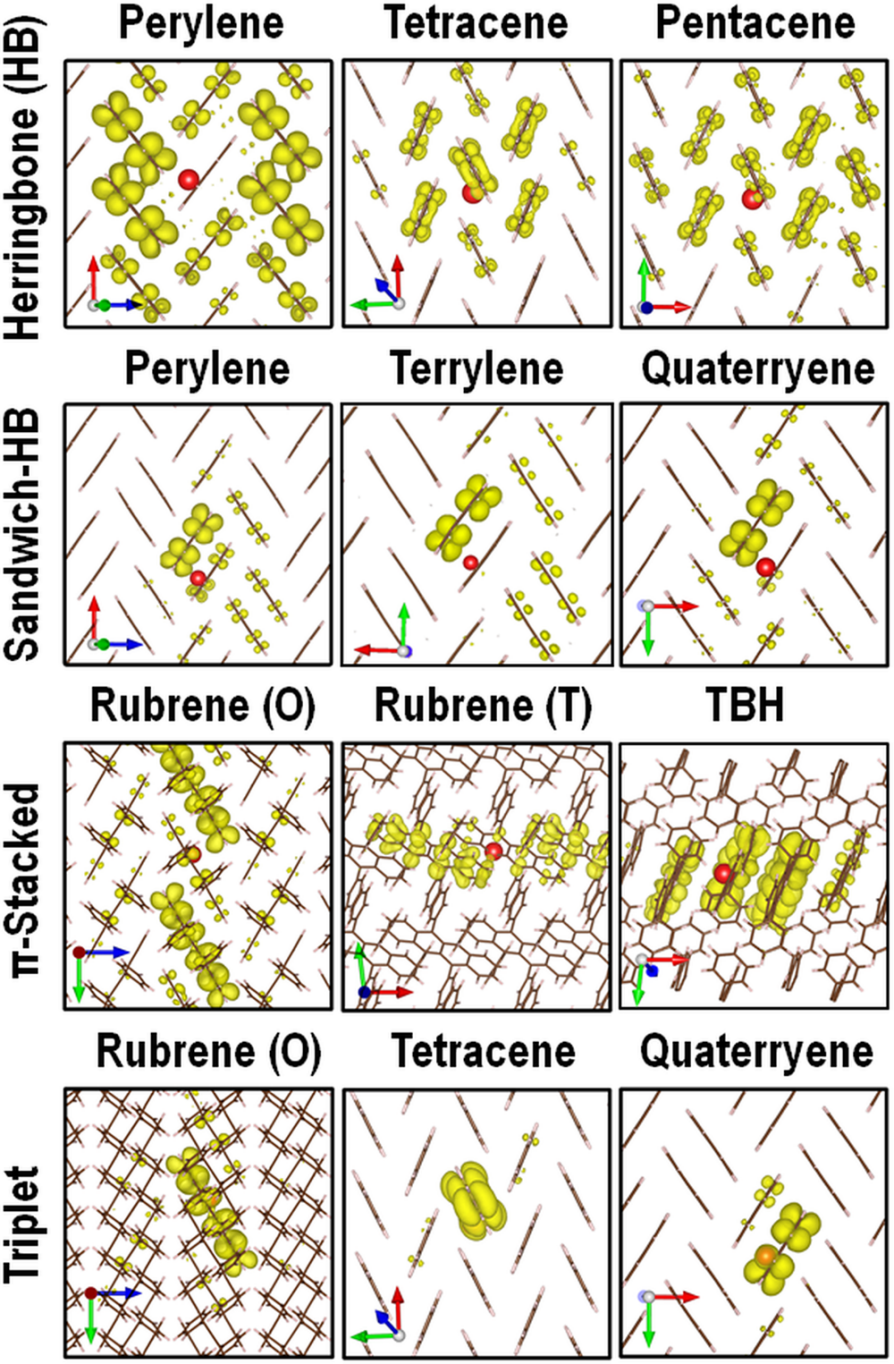
Singlet exciton wave functions characteristic of herringbone (HB), sandwich herringbone (SHB), and π-stacked crystal packing motifs and representative triplet exciton wave functions. The red sphere indicates the hole position and the corresponding electron probability distribution is shown in yellow. For perylene the HB and SHB polymorphs are shown. For rubrene the orthorhombic (O) and triclinic (T) polymorphs are shown. TBH is 9,11,16,18-tetraphenyldibenzo[*de*,*yz*]hexacene (CSD reference code CARREU). The exciton wave functions of perylene, tetracene, pentacene, quaterrylene, and rubrene are reproduced with permission from ref (99). Copyright 2018 AIP Publishing.

Singlet excitons in molecular crystals may be very extended and distributed over many molecules.^99,114,116,148,149,213,214^ The packing of molecules in crystals strongly affects the spatial distribution of singlet excitons.^99,113,114,116^ The electron probability distribution of an exciton is typically localized mainly on the molecules that have the strongest electronic coupling with the molecule on which the hole resides. We have observed qualitative trends in the characteristic form of singlet exciton wave functions in crystals with certain packing motifs, as shown in Figure 3. In crystals with herringbone (HB) packing, the electron probability distribution of the *S*_1_ state is delocalized over several neighboring molecules within a layer that have strong electronic coupling with the molecule, on which the hole resides. In crystals with a sandwich herringbone (SHB) packing the electron probability is typically concentrated predominantly on the dimeric neighbor of the molecule on which the hole is located. In crystals with a π-stacking motif, the electron is distributed mainly on the two nearest neighbors of the molecule with the hole along the π-stacking direction. The crystal packing appears to be more dominant in determining the exciton distribution than the molecular structure. For example, in the HB and SHB polymorphs of perylene the singlet exciton wave function assumes the characteristic form for each packing motif. Triplet excitons tend to be more localized and have a more Frenkel-like character than singlet excitons, regardless of the packing motif.^113,114,212^

The EWF distribution may play a role in SF. It has been suggested that an intermediate intermolecular CT state may be involved in SF; hence, the degree of CT character of the singlet excitation may affect the coupling between *S*_1_ and the multiexciton state of a correlated triplet exciton pair with an overall singlet spin multiplicity.^70,121,215−219^ A singlet exciton with a high degree of CT character would therefore facilitate SF. In addition, the spatial distribution of the exciton wave function determines the entropic contribution to the SF driving force, which may enable SF even when the enthalpic contribution (eq 2) is endothermic.^70,74,88,220−222^ It has been suggested that the entropy gain stems from the number of possibilities for the conversion of *S*_1_ into a correlated pair of triplet excitons, ^1^(*TT*), localized on two molecules within the distribution region of the initial *S*_1_ EWF. Thus, the entropy gain increases with the spatial extent of *S*_1_.^88,220^ A quantitative description of the spatial distribution of the exciton wave function is important for quantifying the CT character of *S*_1_ and calculating the entropy driving force.

**Figure 4:**
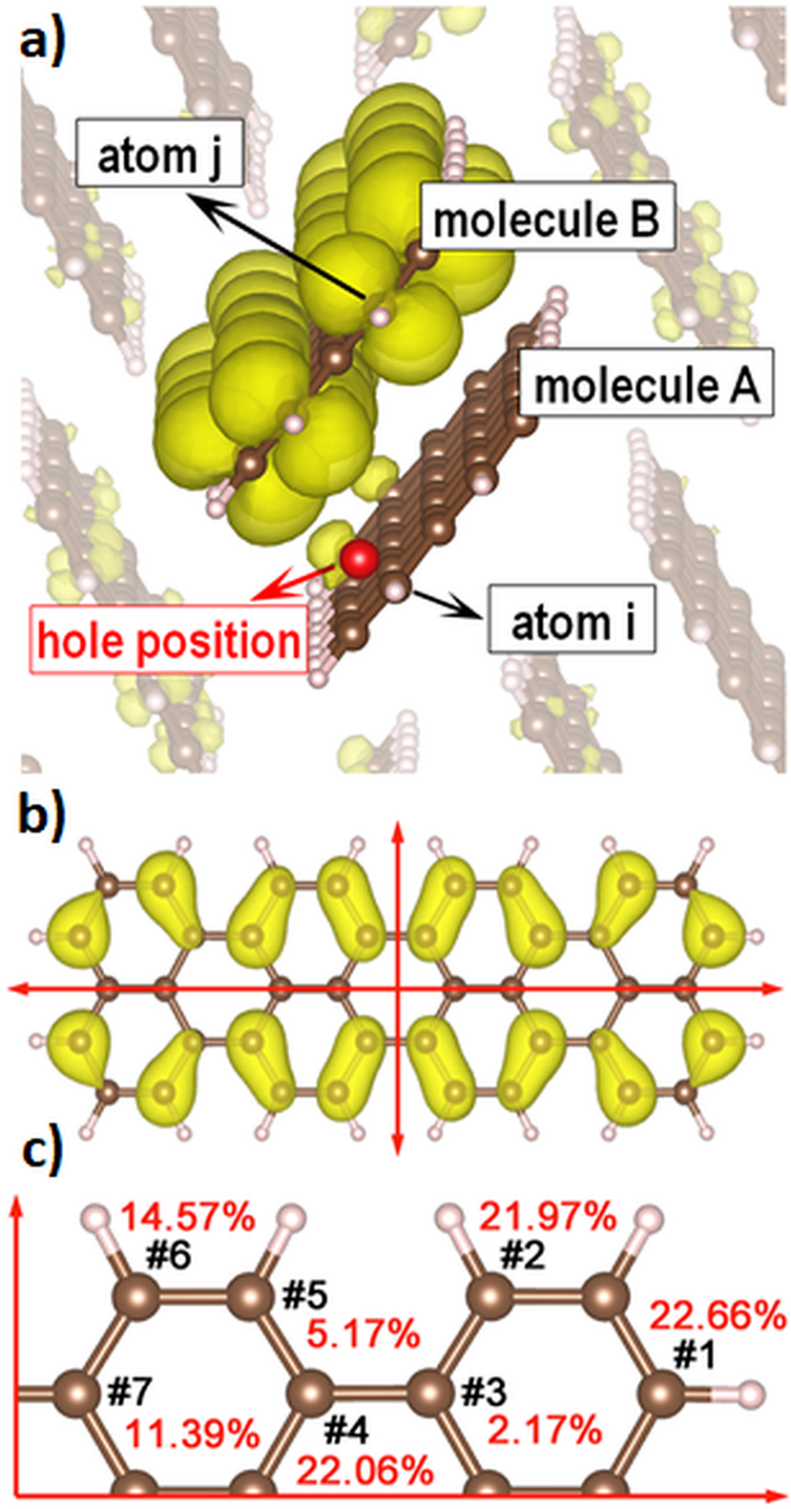
Double-Bader analysis illustrated for quaterrylene: (a) The *S*_1_ exciton wave function of crystalline quaterrylene.^99^ The red dot represents the fixed hole position and the corresponding electron distribution is shown in yellow. (b) The DFT HOMO of quaterrylene. (c) Relative probabilities of finding the hole on all the nonequivalent atoms in the quaterrylene molecule (only one quadrant is shown because of the molecular symmetry), obtained from Bader analysis of the HOMO charge distribution. Reproduced with permission from ref (99). Copyright 2018 AIP Publishing.

Bader analysis^223,224^ is a widely used partitioning scheme for electron charge densities, ρ(**r**_e_), with one position variable, **r**_e_. The charge density is provided in the format of volumetric data on a discrete three-dimensional spatial grid. For each atom, a Bader volume^224^ is defined, which contains a single electron density maximum and is separated from the Bader volumes of neighboring atoms by a zero flux surface of the gradients of the electron density. The charge of each atom is then evaluated by summing over the electron density contained within its Bader volume. Bader analysis cannot be directly applied to exciton wave functions with two position variables, **r**_e_ and **r**_h_, corresponding to the electron and hole, respectively. Double-Bader analysis (DBA)^99,116^ extends the Bader analysis scheme to exciton wave functions by performing nested sums over electron and hole positions. The probability of finding a hole on molecule A and an electron on molecule B, as illustrated in Figure 4a for quaterrylene, is given by
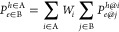
11where *W*_*i*_ is a weight factor corresponding to the relative probability of finding a hole in the Bader volume of atom *i* in molecule A and  is the probability of finding the electron in the Bader volume of atom *j* in molecule B when the hole is located in the Bader volume of atom *i*. Both the hole probability, *W*_*i*_, and the electron probability, , are calculated using Bader analysis. The probability of finding an electron on atom *j* of molecule B when the hole position is fixed in the Bader volume of atom *i* of molecule A, , is obtained via Bader analysis of the BSE exciton wave function, given in eq 9.

The inner sum in eq 11 is then performed over all the atoms of molecule B. The outer sum in eq 11 runs over all the possible hole positions on the atoms of molecule A. To find the weights, *W*_*i*_, Bader analysis is performed on the DFT electron density of the molecular orbital(s), from which the excitation in question originates. The low-lying excited states, such as *S*_1_ and *T*_1_, typically correspond to transitions from the top valence bands to the bottom conduction bands, derived from the single molecule highest occupied molecular orbital (HOMO) and lowest unoccupied molecular orbital (LUMO), respectively. For example, the *S*_1_ excitation of crystalline quaterrylene is about 99% HOMO → LUMO transition. Therefore, the electron charge density distribution of the single molecule HOMO is a reasonable estimate for the relative hole probability. Bader analysis of the DFT HOMO of a single molecule is performed to determine the relative probability of finding the hole on each atom *i* of molecule A, *W*_*i*_, with *∑*_*i*∈A_*W*_*i*_ = 100%. An example of the results for quaterrylene is shown in Figure 4c. The percentage of CT character, %CT, is then defined as the probability of not finding the electron and hole on the same molecule:^99,116^
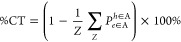
12where *Z* is the number of molecules in the unit cell. For pentacene, the %CT of 97% obtained with DBA agrees with the result of 94% obtained using a different method of evaluation within BSE.^149^ Using different definitions of the charge transfer character may lead to significantly different values.^225^

**Figure 5:**
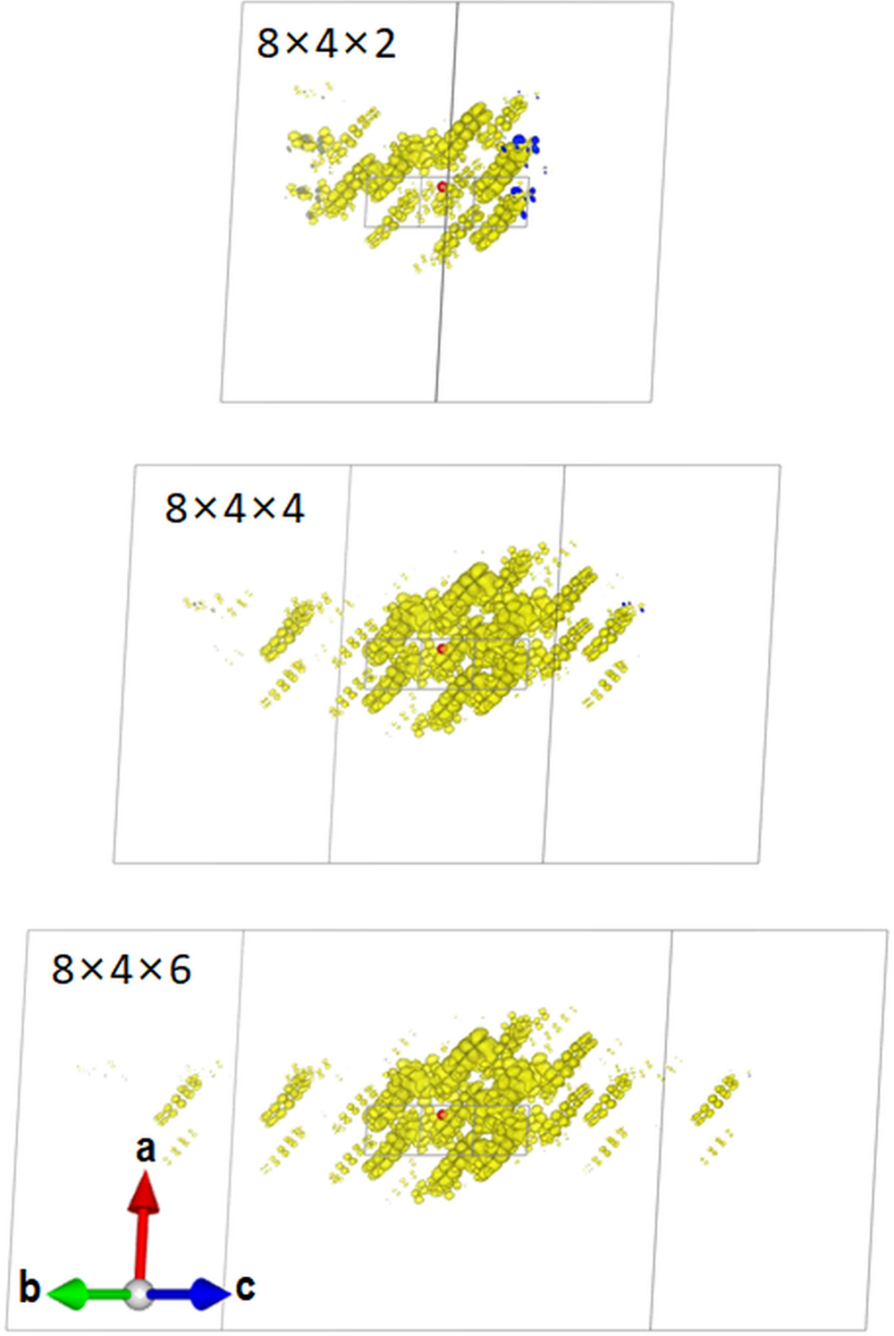
Exciton wave function of TBH (CSD reference code CARREU) visualized for different supercell sizes. The hole position is indicated by the red sphere. The electron probability distribution is visualized in yellow, and its intersections with the supercell boundary are shown in blue.

When performing quantitative analysis of EWFs, it is important to ensure that they are properly converged. In the BerkeleyGW code,^167^ the fine *k*-point grid corresponds to the supercell used to calculate the EWF. As shown in Figure 3, EWFs can extend over many unit cells. Therefore, to achieve convergence the fine grid should be set, such that the EWF is fully contained within the supercell.^116^ This is visualized for 9,11,16,18-tetraphenyldibenzo[*de*,*yz*]hexacene (TBH, CSD reference code CARREU) in Figure 5. This is a representative example of π-stacked molecular crystals, in which the EWFs typically extend along the stacking direction, requiring a larger number of *k*-points along the corresponding direction in reciprocal space. With a fine grid of 8 × 4 × 2, the EWF intersects with the supercell boundary. With a a fine grid of 8 × 4 × 6, the EWF is fully contained within the unit cell.

**Figure 6:**
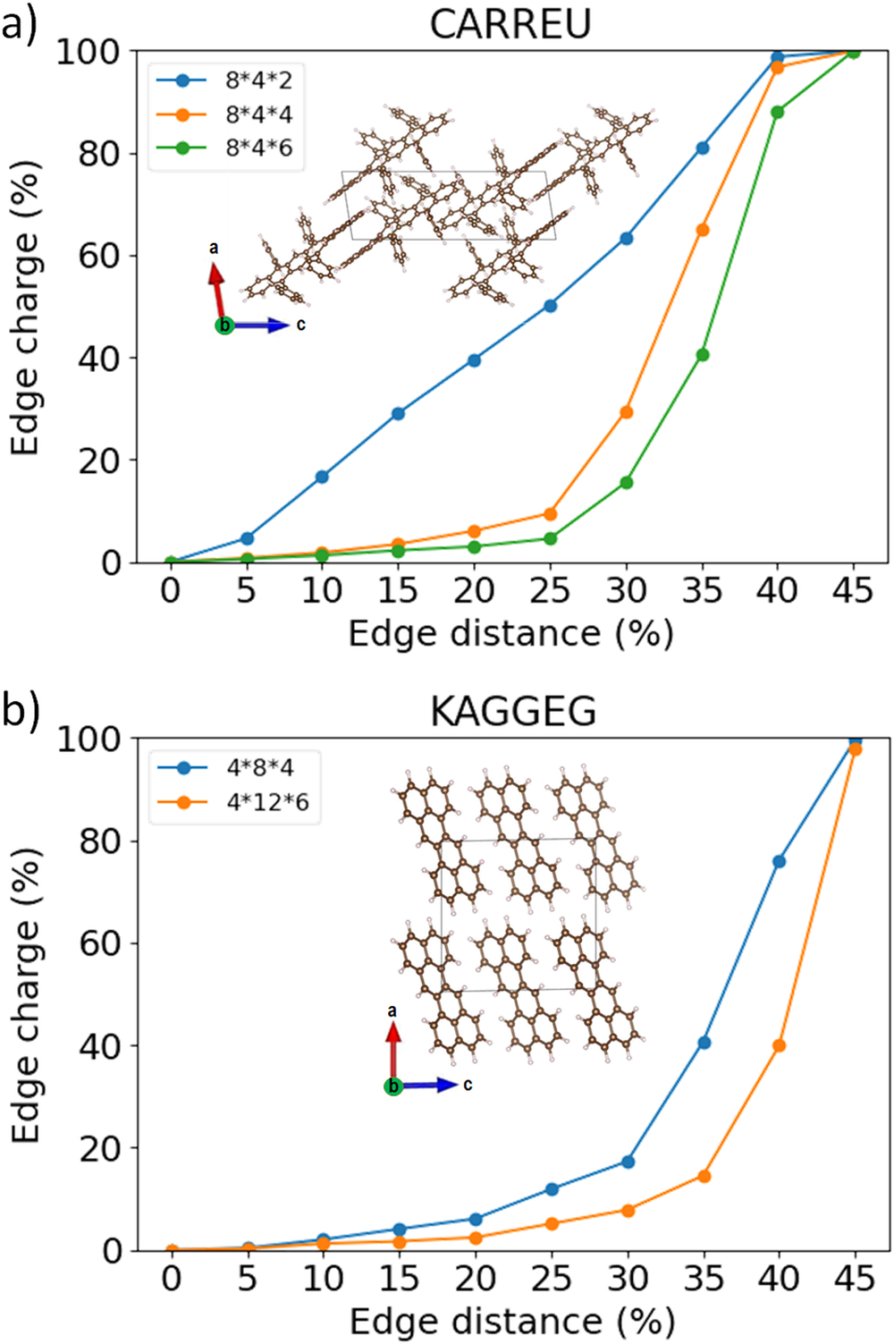
Exciton wave function convergence as indicated by edge charge as a function of edge distance with different supercell sizes for (a) 9,11,16,18-tetraphenyldibenzo[*de*,*yz*]hexacene (CSD reference code CARREU) and (b) dibenzo[*de*,*mn*]naphthacene (CSD reference code KAGGEG).

To quantify whether the EWF is fully contained in the supercell, we define the edge distance, *d*, as a fraction of the lattice parameters. The corresponding fractional volume of the central region of the supercell is given by
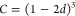
13The edge region is the part of the supercell outside of this central region. To calculate the edge charge, Bader analysis is performed for the electron density with the hole position fixed on a molecule in the central subcell of the supercell. The fraction of edge charge is evaluated by summing over the Bader charges on the atoms in the edge region and dividing by the total charge contained in the supercell. We consider the supercell size converged if the edge charge at an edge distance of 25% is less than 5%. This means that over 95% of the electron density is contained within a central region of 12.5% of the supercell volume. The EWF convergence is demonstrated in Figure 6 for TBH and dibenzo[*de*,*mn*]naphthacene (Z-P, CSD reference code KAGGEG). Based on eq 13, when *d* is 45%, *C* is only 0.1%. This leads to the sharp increase of the edge charge to 100% as the edge distance approaches 50%. For TBH, the edge charge at an edge distance of 25% is 50% with a fine grid of 8 × 4 × 2, 9.5% with a fine grid of 8 × 4 × 4, and 4.6% with a fine grid of 8 × 4 × 6, at which point the EWF is converged. For Z-P, the edge charge at an edge distance of 25% decreases from 12% with a fine grid of 4 × 8 × 4 to 5% with a fine grid of 4 × 12 × 6. The EWF convergence procedure can be performed automatically using the Python program dbaAutomator.^116^

### GW+BSE Assessment of Candidate SF Materials

**Figure 7:**
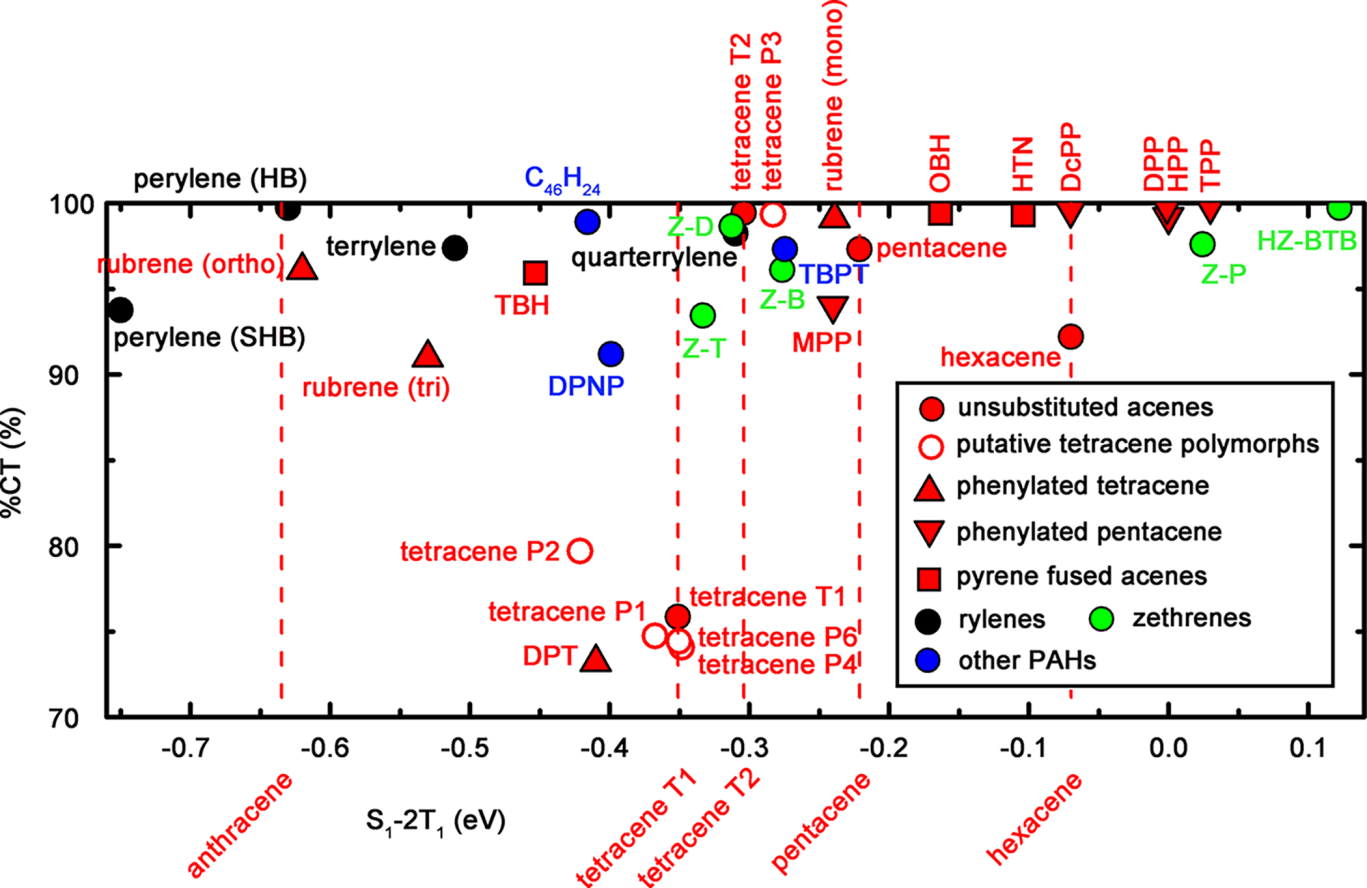
Candidate SF materials assessed with respect to a two-dimensional descriptor calculated with GW+BSE.^99,100,113−119^ The thermodynamic driving force for SF (*S*_1_ – 2*T*_1_) is displayed on the *x*-axis, and the singlet exciton charge transfer character (%CT) is displayed on the *y*-axis. Acenes and their derivatives are colored in red, rylenes in black, zethrenes in green, and other PAHs in blue. The *S*_1_ – 2*T*_1_ values of unsubstituted acenes are indicated by vertical dashed lines. The corresponding molecular structures are shown in Figure 8.

To assess the likelihood of candidate molecular crystals to undergo SF based on GW+BSE calculations, we have proposed a two-dimensional descriptor for SF efficiency,^99^ shown in Figure 7. The primary descriptor, displayed on the *x*-axis, is the energetic driving force for SF, *S*_1_ – 2*T*_1_ (eq 2). The secondary descriptor (explained and justified above), displayed on the *y*-axis, is the degree of charge-transfer character of the lowest singlet exciton, %CT (eq 12). The aforementioned limitations of G_0_W_0_+BSE@PBE lead to a systematic underestimation of the singlet and triplet excitation energies.^26^ As a result, the values of the SF driving force in Figure 7 are always too negative. Considering this, the accuracy of G_0_W_0_+BSE@PBE is adequate for *comparative assessment* of different materials.

Acenes and their derivatives are arguably the most well-studied SF materials.^71,114,148,226^ The SF driving force values of unsubstituted acenes are indicated by the dashed lines in Figure 7. The SF driving force increases across the acene series. Anthracene (not shown in the plot because its %CT is below 70%) and its derivatives are typically TTA chromophores.^26^ In the common form of tetracene, labeled T1, SF is slightly endothermic with an experimentally measured driving force of −0.21 eV.^72^ However, SF may still occur in a material with a slightly endothermic driving force if the entropic contribution is large enough.^74,75^ The thin film form of tetracene, labeled T2, has been observed to undergo significantly faster SF than the common form.^227^ In pentacene, SF is slightly exothermic with an experimentally measured driving force of 0.11 eV.^72^ The GW+BSE SF driving force of hexacene is higher than that of pentacene, which may make the energy loss in the SF pathway too high. SF in hexacene has been found experimentally to have a time scale of 530 fs, which is orders of magnitude faster than tetracene (10–100 ps) but significantly slower than pentacene (80–110 fs).^228,229^ This has been attributed to a multiphonon relaxation effect originating from a larger exothermicity than pentacene.^228^ The trend in the SF driving force values across the acene series is in agreement with these experimental observations as well as other computational studies.^73,230^ The degree of charge transfer character is not experimentally measurable; however, the materials experimentally observed to undergo efficient SF, such as pentacene and tetracene T2, have a high %CT. In addition, the lower %CT of hexacene compared to pentacene is consistent with the slower SF in hexacene despite its higher driving force.

Of the materials experimentally known to undergo SF, displayed in Figure 7, the common orthorhombic polymorph of rubrene has the lowest GW+BSE SF driving force, only slightly higher than anthracene. This is consistent with the experimental observation that rubrene may exhibit either TTA or SF.^94−96^ Below orthorhombic rubrene, we find the two polymorphs of perylene, a known TTA material.^26^ Another experimentally known SF material, the tetracene derivative DPT^49,231^ has a GW+BSE SF driving force and %CT close to tetracene. (We note, however, that SF was experimentally observed in amorphous films of DPT, not in the crystal structure studied here.) Therefore, we consider materials in the range between orthorhombic rubrene and tetracene as possible SF candidates, but perhaps SF in these materials would be slower and/or have a lower quantum yield than in pentacene. We consider materials with higher GW+BSE SF driving force values than tetracene as likely to undergo SF. Another experimentally known SF material, the pentacene derivative DPP,^232^ has a higher GW+BSE SF driving force than pentacene and hexacene. Materials whose GW+BSE SF driving force is between tetracene and pentacene are considered as the best candidates because they are likely to undergo efficient SF with a moderate energy loss.

**Figure 8:**
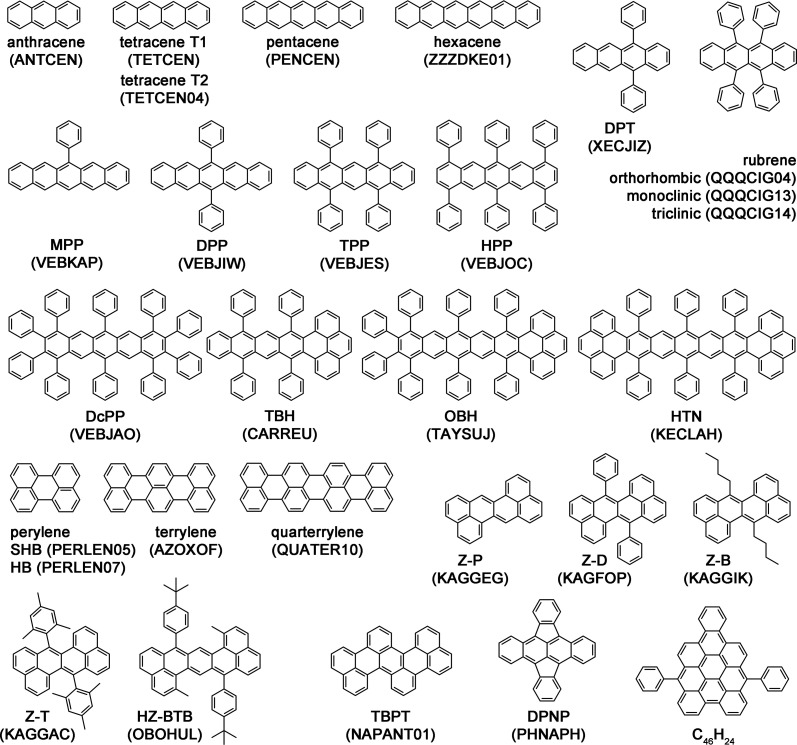
Molecular structures of the materials displayed in Figure 7 with their Cambridge Structural Database (CSD) reference codes shown in parentheses: anthracene, tetracene, pentacene, hexacene, 5,12-diphenyltetracene (DPT), rubrene, 6-phenylpentacene (MPP), 6,13-diphenylpentacene (DPP), 5,7,12,14-tetraphenylpentacene (TPP), 1,4,6,8,11,13-hexaphenylpentacene (HPP), 1,2,3,4,6,8,9,10,11,13-decaphenylpentacene (DcPP), 5,7,14,16-tetraphenyl-8:9,12:13-bisbenzohexatwistacene (TBH), 1,2,3,4,6, 8,15,17- octaphenyl-9:10,13:14-bisbenzoheptatwistacene (OBH), 6,8,10,17,19,21-hexaphenyl-1.22,4.5,11.12,15.16-tetrabenzononatwistacene (HTN), perylene, terrylene, quarterrylene, dibenzo[*de*,*mn*]naphthacene (Z-P), 7,14-diphenyldibenzo[*de*,*mn*]naphthacene (Z-D), 7,14-di-*n*-butyldibenzo[*de*,*mn*]naphthacene (Z-B), 14-bis(2,4,6-trimethylphenyl)dibenzo[*de*,*mn*]naphthacene (Z-T), 7,15-bis(4-*tert*-butylphenyl)-1,9-dimethylheptazethrene (HZ-BTB), tetrabenzo[*de*,*hi*,*op*,*st*]pentacene (TBPT), 5,6,11,12-tetraphenynaphthacene (DPNP), and 7,14-diphenylnaphtho[1,2,3,4-*cde*]bisanthene (C_46_H_24_).

With the above criteria in mind, we have assessed several materials, whose molecular structures are shown in Figure 8, as potential SF candidates. This includes the lesser known polymorphs of rubrene,^113^ phenylated acenes,^114^ pyrene-fused acenes,^116^ rylenes,^99,100^ zethrenes,^115^ and other polycyclic aromatic hydrocarbons (PAHs).^117,118^ The excitonic properties of molecular crystals are determined by both the single molecule properties and the crystal packing. Of the materials displayed in Figure 7, three have known polymorphs: rubrene, perylene, and tetracene. It has been experimentally observed that crystal packing can significantly affect the SF performance.^227,233,234^ Indeed, significant differences are found here between the SF driving force and the exciton distributions of different polymorphs. At the GW level, the crystal packing affects the dielectric screening and band dispersion, which may change the band structure and fundamental band gap.^113,114^ This is also reflected in changes in the BSE *S*_1_ and *T*_1_ energies. Although these changes are only fractions of an eV, they can be decisive for whether or not a material will undergo SF, as well as for the rate and quantum yield of the SF process, in particular for materials like tetracene whose SF driving force is marginal. We note that the entire range of SF driving force values displayed in Figure 7 is less than an eV. For example, the GW+BSE SF driving force of monoclinic rubrene is a little lower than that of pentacene, placing it in the range we consider ideal, in contrast to the common orthorhombic polymorph.^113^

Functionalization of a parent compound with side groups can directly affect the molecular excitation energies^26,114,116^ as well as indirectly affect the molecular crystal’s excitation energies by modifying the crystal packing.^114,116,222^ Much of the development of new SF materials has focused on derivatives of known compounds.^82,145,235,236^ Of the phenylated acenes displayed in Figure 7, MPP has *S*_1_ – 2*T*_1_ and %CT slightly lower than pentacene, placing it in the ideal range for SF. The phenylated pentacene derivatives DcPP, HPP, and TPP have a GW+BSE SF driving force greater than or equal to hexacene and a higher %CT than hexacene. For these materials, the energy loss in SF may be too high for practical applications. Functionalization with side groups may affect additional molecular properties, such as stability. The poor stability of unsubstituted acenes presents a challenge for device applications. One strategy for stabilizing acenes is attaching pyrene to either or both ends. Of the pyrene-fused acenes displayed in Figure 7, TBH has a lower SF driving force than tetracene, whereas OBH and HTN have a somewhat higher SF driving force than pentacene but lower than hexacene.^116^ We note that around the time our theoretical study was published, an experimental paper was published reporting SF in pyrene-fused N-substituted tetracene derivatives (in solution),^237^ and subsequently, another experimental report was published of SF in the solid state in a related stabilized acene derivative.^238^ Very recently, films of pyrene-fused N-substituted acene derivatives have been reported to exhibit ultrafast SF as well as excellent stability.^239^ These experiments confirm our prediction of pyrene-fused acene derivatives as promising SF materials.

Computer simulations are useful for investigating materials from underexplored or new chemical families.^77^ As shown in Figure 7, the rylene family exhibits a similar trend to the acene family of increasing SF driving force with increasing backbone length. Perylene is more likely to undergo TTA than SF. Terrylene, whose crystal structure was solved only recently,^100^ is in the range between orthorhombic rubrene and tetracene, where SF is possible. Quaterrylene has an optimal SF driving force between tetracene and pentacene and is also more stable than acenes.^99^ The unsubstituted zethrene, labeled Z-P in Figure 7, has a higher SF driving force than hexacene, close to the phenylated pentacene derivatives DPP, HPP, and DPP. The three zethrene derivatives, labeled Z-T, Z-D, and Z-B, are concentrated in the optimal region of the two-dimensional descriptor between tetracene and pentacene. This demonstrates again that chemical functionalization is an effective means of fine-tuning SF energetics by modifying the isolated molecule properties and the crystal packing (as well as potentially contributing to the chemical stabilization of some compounds). The zethrene family also shows a trend of increasing SF driving force with molecule size. HZ-BTB, which has a longer backbone, has a higher SF driving force than all the smaller zethrene derivatives.^115^ The three PAH molecules shown in blue in Figure 7 demonstrate how computational exploration may accelerate the discovery of new classes of SF materials. C_46_H_24_ is a phenylated graphene flake whose crystal structure was recently solved.^118^ TBPT, which has a system of fused benzene rings reminiscent of rylenes, and DPNP, which has a mixed system of fused 5- and 6-membered rings, were discovered through exploration of a set of 101 PAHs whose crystal structures are available in the CSD.^117^ Of these materials, C_46_H_24_ and DPNP are in the range between orthorhombic rubrene and tetracene, where SF is possible. TBPT is in the ideal range between tetracene and pentacene.

**Figure 9:**
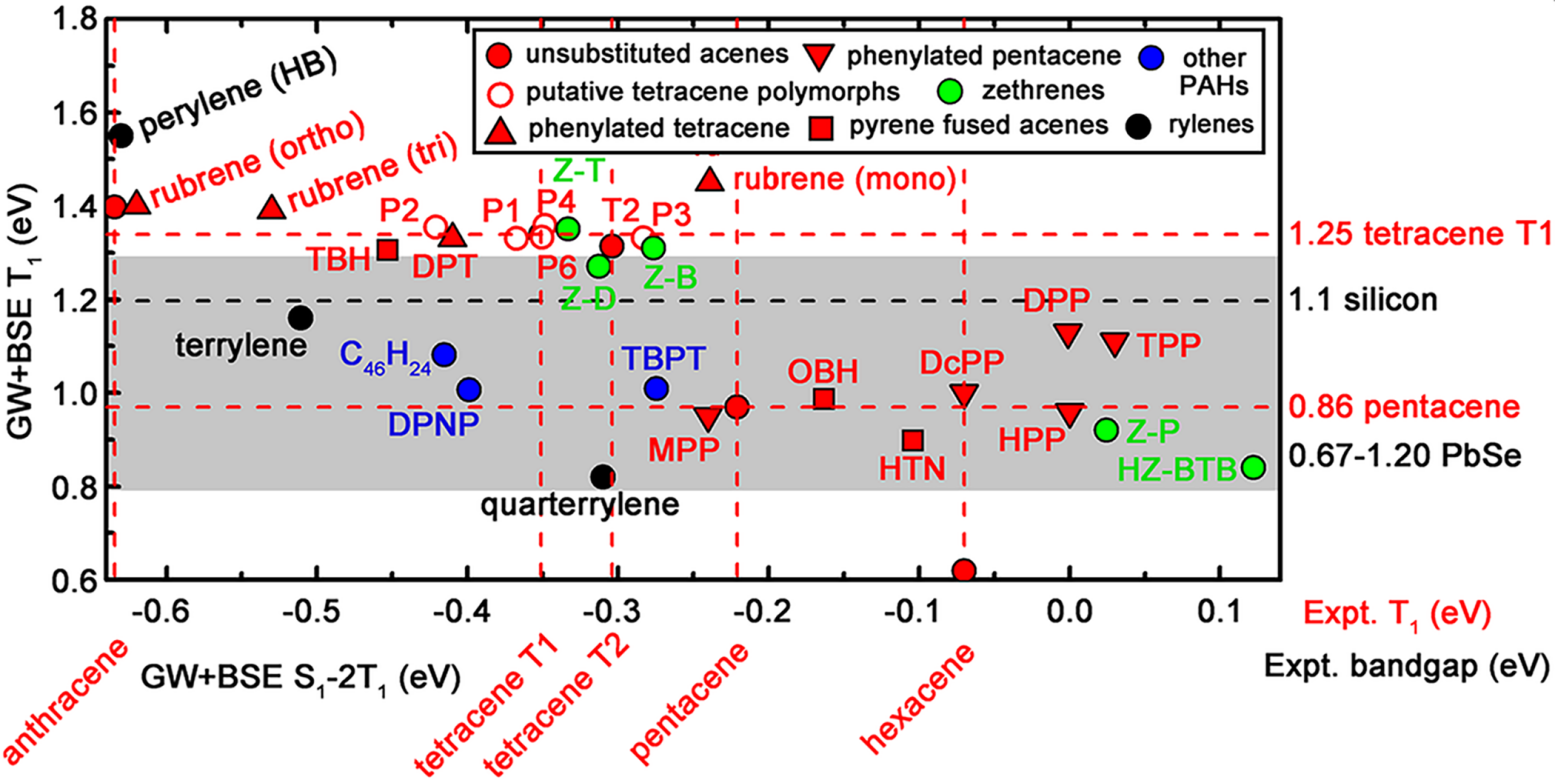
GW+BSE *T*_1_ on the *y*-axis as a function of the GW+BSE *S*_1_ – 2*T*_1_ on the *x*-axis for candidate SF materials. Acenes and their derivatives are colored in red, rylenes in black, zethrenes in green, and other PAHs in blue. The GW+BSE *T*_1_ values of tetracene T1 and pentacene, 1.34 and 0.97 eV, are indicated by red horizontal dashed lines. Their corresponding experimental values, 1.25 and 0.86 eV, are shown on the right. Experimental band gaps of silicon and PbSe nanocrystals, indicated by a black horizontal dashed line and a shaded area, are placed in the plot with respect to experimental *T*_1_ values of tetracene T1 and pentacene.

Once a candidate SF material is identified with respect to *S*_1_ – 2*T*_1_ and %CT in Figure 7, it needs to be paired with complementary solar cell materials for triplet harvesting. Figure 9 shows the *T*_1_ excitation energies of candidate SF materials compared with the band gaps of representative photovoltaic materials. (We note, however, that excitation energies may be renormalized due to the interactions at an interface.^240^) For solar cells based on materials with larger band gaps, such as GaAs (1.42 eV), CdTe (1.45 eV), and most perovskites, it is hard to find complementary SF materials because the *T*_1_ excitation energies of typical SF materials are lower than the band gaps of these absorbers. Moreover, with increasing absorber band gap, the transmission loss becomes dominant, rather than the thermalization loss. This means that such solar cells stand to benefit more from TTA upconversion than from SF. The *T*_1_ energy of the common form of tetracene (1.25 eV, indicated by the higher horizontal dashed line in Figure 9) is slightly higher than the band gap of silicon (indicated by the black horizontal dashed line in Figure 9), which facilitates pairing with commercial Si cell technology. However, as discussed above, tetracene is not the best performing SF material and is not sufficiently stable. Therefore, alternative SF materials with a close *T*_1_ energy to tetracene are needed. Of the materials displayed in Figure 9, the putative polymorphs of tetracene and the tetracene derivatives DPT, rubrene, and TBH have *T*_1_ excitation energies close to tetracene. Of the compounds with no relation to tetracene, three zethrene derivatives, Z-T, Z-D, and Z-B, have *T*_1_ energies close to tetracene. Hence, these candidate SF materials would pair well with silicon and other materials with similar band gaps, such as Sb_2_Se_3_ (1.1 eV), Cu_2_ZnSn(S,Se)_4_ (1.0–1.5 eV), and Cu(In,Ga)Se_2_ (1.02–1.67 eV).^241,242^

**Figure 10:**
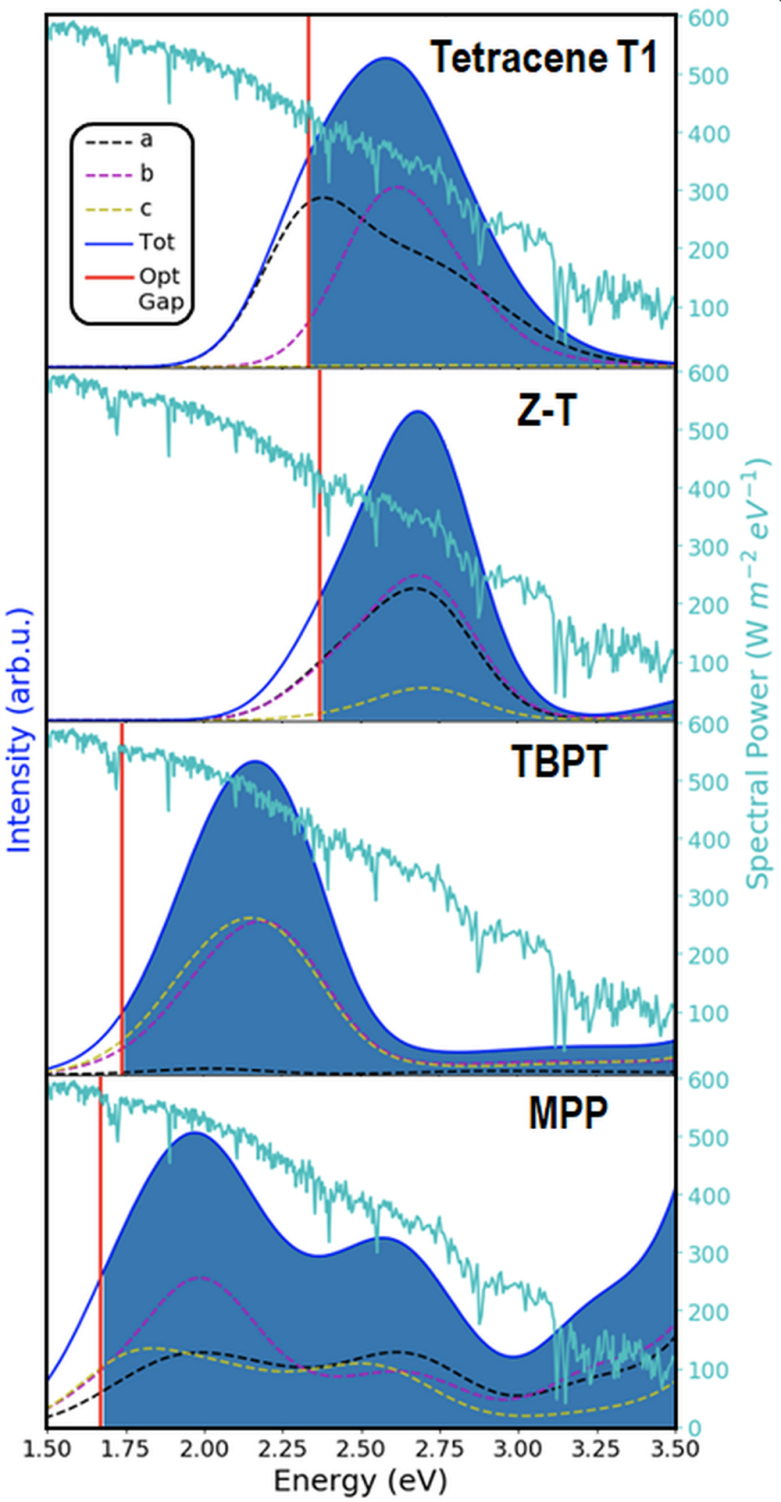
GW+BSE@PBE absorption spectra of the common form of tetracene (T1), the zethrene derivative Z-T, TBPT, and the phenylated pentacene MPP. The red vertical lines indicate the optical gaps. The solar spectrum is also shown.

SF materials whose *T*_1_ excitation energies are smaller than the band gap of silicon, such as pentacene (0.86 eV, indicated by the lower horizontal dashed line in Figure 9), can be paired with quantum dots, whose band gap can be tuned by changing the particle size. For example, the band gap of PbSe nanocrystals ranges from 0.67 to 1.20 eV (indicated by the shaded gray area in Figure 9).^51^ Many SF candidates have *T*_1_ excitation energies in that range including pentacene derivatives, larger rylenes, some zethrenes, and other PAHs. An advantage of SF materials and absorbers with narrower gaps is that they can absorb more of the solar spectrum (see also Figure 10). In addition to pairing with compatible inorganic absorbers, candidate SF materials may be used in organic photovoltaic devices. Organic photovoltaic devices consist of a heterojunction of an electron donor material and an electron acceptor material. At the interface, excitons dissociate into electrons in the LUMO of the acceptor and holes in the HOMO of the donor. In such architectures, the SF material typically functions as a donor and is paired with an acceptor to extract triplet excitons as well as an additional absorber to harvest photons with energies below the *S*_1_ energy of the SF material.^46,232,243^ In this respect, GW may be used to predict the energy level alignment at the donor–acceptor interface.^244,245^

Another consideration in the selection of SF materials is that they should absorb light strongly at the optical gap (which corresponds to *S*_1_) so a thin layer of material would be sufficient, as discussed above. GW+BSE can be used to calculate absorption spectra. In the absence of information on the polarization of light used in an experiment, it is customary to sum over the absorption with light polarized along the three crystal axes.^246^ One of the advantages of tetracene, shown in Figure 10, and pentacene^113,246^ is a prominent absorption peak at the optical gap. Of the materials considered here as candidates to replace tetracene based on their excitation energies, the thin film form of tetracene T2 and the putative polymorph P3 retain this feature^119^ as well as the monoclinic form of rubrene.^113^ Of the zethrene derivatives whose excitation energies are close to tetracene, Z-T, shown in Figure 10, has the strongest absorption at the optical gap.^115^ Of the materials considered here as candidates to replace pentacene, TBPT and MPP have an absorption peak at the optical gap, as shown in Figure 10. MPP has a relatively narrow gap and a broad absorption peak enabling it to harvest a larger swath of the solar spectrum.

### Combining GW+BSE with ML and Optimization

The search for SF materials includes exploring the chemical compound space and optimizing the crystal packing of candidate molecules. Therefore, there is still tremendous potential for new discoveries. Owing to the high computational cost of GW+BSE, this approach can only be used for detailed studies of a small number of candidate materials in the final stages of a hierarchical workflow, in which models that are fast to evaluate are used for prescreening of a large number of candidates. Such models may be physical/chemical models, such as the multiradical character,^247^ or machine learned (ML) models. The vast majority of chemical space exploration efforts have focused on isolated molecules.^68,88,136−139,248−253^ Here, we discuss two examples focused on crystalline materials.

#### Genetic Algorithm Optimization of Crystal Packing

The first example is optimizing the crystal packing of tetracene to enhance its SF performance.^119^ As explained above, SF in the common bulk crystal form of tetracene (labeled T1) is slightly endoergic,^72^ and it has been observed experimentally that a thin film polymorph (labeled T2) has a significantly better performance.^227^ This raises the question of whether a more optimal crystal packing can be achieved. A putative polymorph predicted to have improved SF performance would also have to be sufficiently stable to be synthesizable. Crystal structure prediction (CSP) methods aim to explore the configuration space of all the possible crystal structures of a given molecule by computer simulations and find its most likely crystal structure(s).^254^

To perform CSP, we used the genetic algorithm (GA) code GAtor^255,256^ and its associated random structure generator, Genarris.^257^ GAs rely on the evolutionary principle of survival of the fittest to perform global optimization. The target property is mapped onto a fitness function, and structures with a high fitness have an increased probability to “mate” by combining their structural “genes” to create offspring. The process repeats iteratively until an optimum is found. For CSP, an energy-based fitness function is typically used. Lower energy corresponds to higher stability and therefore to higher fitness. However, GA fitness functions can be tailored to optimize any property of interest. We formulated a fitness function designed to simultaneously optimize the stability and SF performance with equal weights.^119^ The energy term of the fitness function was based on dispersion-inclusive DFT,^255^ using the PBE functional and the Tkatchenko-Scheffler (TS) dispersion correction.^800^ It is unfeasible to use GW+BSE for the SF term of the fitness function, owing to the high computational cost. Instead, we have chosen a descriptor that is fast to evaluate.

Michl et al. developed a simplified dimer-based model for evaluating SF rates, implemented in the Simple code.^258,259^ In the SF process, the singlet exciton on the excited chromophore is converted into a biexciton state of two correlated triplet excitons with an overall singlet spin, which then dissociates into two separate triplet excitons. Simple assumes that the formation of the biexciton state is the rate-determining step and computes the rate of biexciton formation. The SF electronic matrix elements are evaluated on the basis of a frontier orbital model using only the highest occupied molecular orbital (HOMO) and the lowest unoccupied molecular orbital (LUMO) of the two molecules and two electrons from each molecule. This results in a simplistic four-electron four-orbital space with the rest of the electrons forming a fixed core. This model is further simplified by truncating it to a few singlet configurations, and a series of approximations are introduced for qualitative evaluation of the dimer geometries. Finally, the SF rate of a dimer is calculated using the Marcus rate theory.^258^ The contribution to the SF rate from the lower exciton state S* is
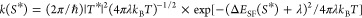
14Here λ is the reorganization energy, Δ*E*_SF_(S*) = *E*(TT*) – *E*(S*), |T*|^2^ is square of the interaction Hamiltonian (*H*_int_) matrix element ⟨S*|*H*_int_|TT*⟩, *T* is the temperature, *k*_B_ is the Boltzmann constant, and *ℏ* is Planck’s constant. *k*(S**) is evaluated using a similar expression. The total SF rate, *k*(SF), is a weighted sum of both the rates given by
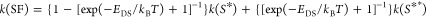
15where *E*_DS_ = *E*(S**) – *E*(S*) is the Davydov splitting. The calculation of the matrix elements using the frontier orbital model, the estimation of the reorganization energy, and the assumptions and numerical approximations used in Simple are described in detail in ref (258). We note that the Simple model can only be used to compare different dimer configurations of the same compound, not to compare between different materials. To evaluate the SF rate of a molecular crystal using Simple, dimers are extracted from the crystal structure. The highest result obtained is taken as the SF rate of that structure and used to evaluate the SF term of the fitness function.

**Figure 11:**
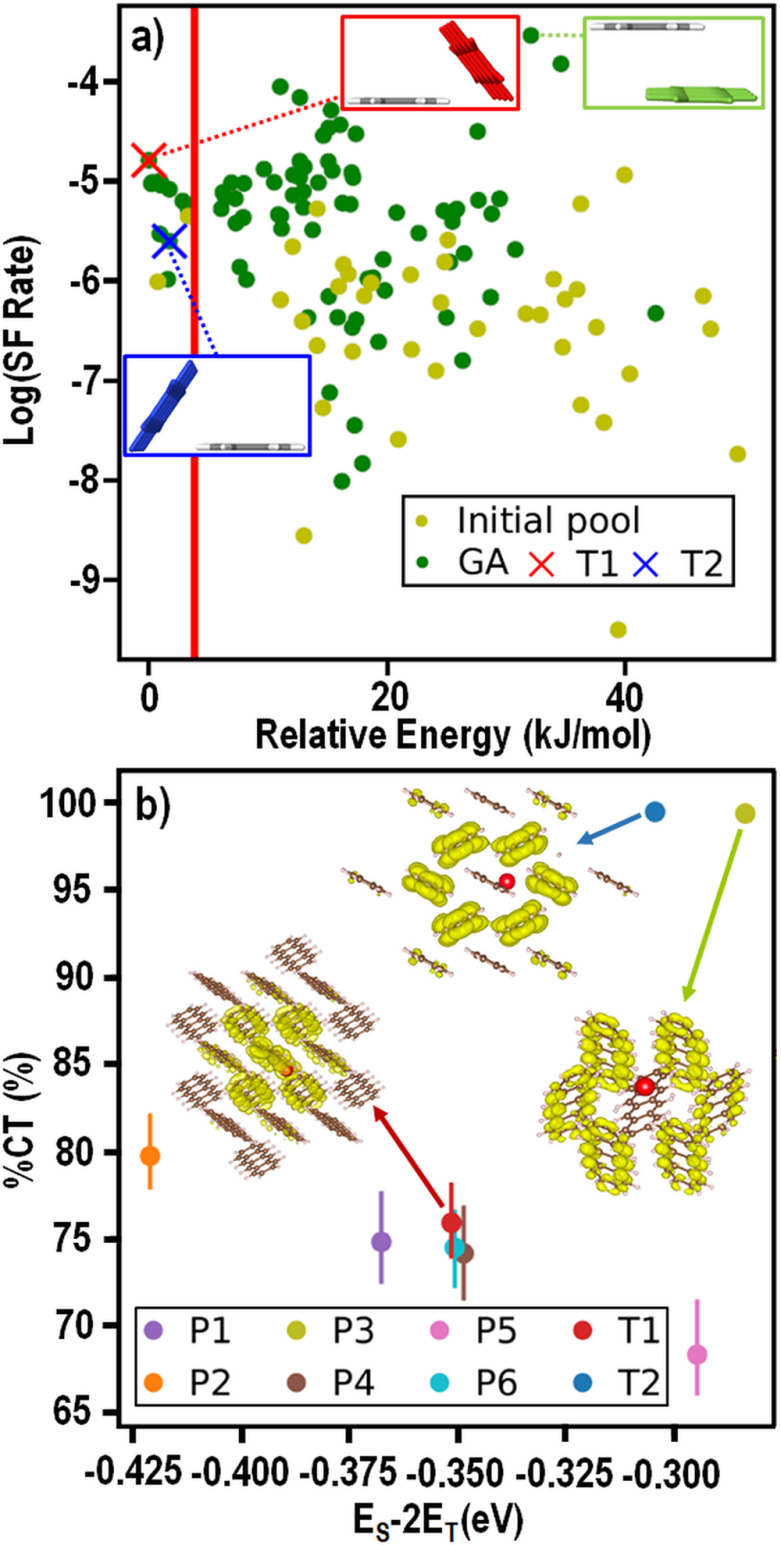
Genetic algorithm (GA) optimization of the crystal packing of tetracene for enhanced SF performance: (a) Logarithm of the Simple SF rate vs the DFT relative energy for putative crystal structures of tetracene. Structures comprising the initial population are colored in light green, and structures generated by the GA are colored in dark green. The two known forms of tetracene are marked by red and blue crosses. The polymorph energy range of 4 kJ/mol is indicated by the red line. Dimers extracted from the structure with the highest Simple SF rate and from the two known forms of tetracene are also shown. The molecule colored in gray is equivalent. (b) GW+BSE evaluation of the structures within the polymorph energy range. The putative structures are labeled in order of stability. The singlet exciton wave functions of the two known forms and the most promising putative structure of tetracene are visualized with the hole position indicated by the red dot and the electron distribution colored in yellow. Reproduced with permission from ref (119).

Figure 11a shows the results of a GAtor run using the tailor-made fitness function that simultaneously minimizes the energy and maximizes the Simple SF rate. The Simple rate is plotted as a function of the relative energy. GAtor successfully generates many crystal structures with an increased SF rate compared to the initial population of random structures generated by Genarris. It is apparent that for tetracene there is a trade-off between stability and SF performance. In the dimer with the highest SF rate (shown in the green box) the two molecules lie parallel to each other with significant cofacial overlap. This packing motif does not seem to be energetically favorable, based on its absence from the low-energy structures, in which packing motifs with the molecules tilted with respect to each other are more common (dimers extracted from the T1 and T2 structures shown in the red and blue boxes, respectively). However, it may be possible to stabilize structures with cofacial backbone packing by side-group functionalization.

Structures within 4 kJ/mol of the T1 form, which is the energy global minimum, were reoptimized with more stringent DFT settings and evaluated with GW+BSE. The results are shown in Figure 11b. It is demonstrated yet again that crystal packing can significantly affect the SF driving force for a given compound as well as the exciton wave function distribution. A putative tetracene polymorph, labeled P3, is found to have a higher SF driving force than both the T1 and T2 forms of tetracene as well as a very high %CT. The putative tetracene structures generated by GAtor are also compared to other materials in Figure 7 (open red circles). The P3 putative structure has a GW+BSE SF driving force between tetracene and pentacene, close to the zethrene derivative Z-B and the other PAH TBPT. The P3 structure is only 1.5 kJ/mol higher in energy than the T1 structure of tetracene, well within the range of experimentally viable polymorphs. Thus, inverse design by a property-based GA is a useful strategy for exploring the crystal structure landscape of candidate SF materials to discover potential polymorphs with improved SF performance.

#### Machine Learning Predictive Models for SF

In the second example, ML is used to generate models that can predict GW+BSE SF driving force values and are faster to evaluate.^117^ These low-cost machine-generated models can then be used for wider exploration of materials, whose crystal structure is known, but have not been previously considered in the context of SF. To this end, the sure-independence-screening-and-sparsifying-operator (SISSO)^260^ ML algorithm was employed. The input of SISSO is a set of primary features, which are physical descriptors that could be correlated to the target property. Physical and chemical insight is applied in the choice of primary features. The primary features used in this case included both single molecule properties and crystal properties, calculated at the DFT (PBE) level. SISSO generates a huge feature space by iteratively combining the primary features using linear and nonlinear algebraic operations with rules to avoid generating unphysical models. Linear regression is performed to identify the best performing models for each dimension and rung, which is the number of times primary features are combined. The higher the dimension and rung, the more complex is the model. Because overly complex models can lead to overfitting, we limited the dimension and rung to 4 and 3, respectively. The resulting models are labeled as *M*_Dim,Rung_. We note that although the primary features are physically motivated, the models generated by SISSO by combining primary features do not necessarily have a physical interpretation. An important advantage of SISSO is that it can work well with a relatively small amount of data.^261−268^ This is critical in this case because of the high computational cost of acquiring GW+BSE training data for molecular crystals. To train SISSO, we generated a data set containing GW+BSE SF driving force values of 101 PAH molecular crystals with up to ∼500 atoms in the unit cell (the PAH101 set). 91 data points were used as the training set, and the remaining 10 were used as an unseen test set.

**Figure 12:**
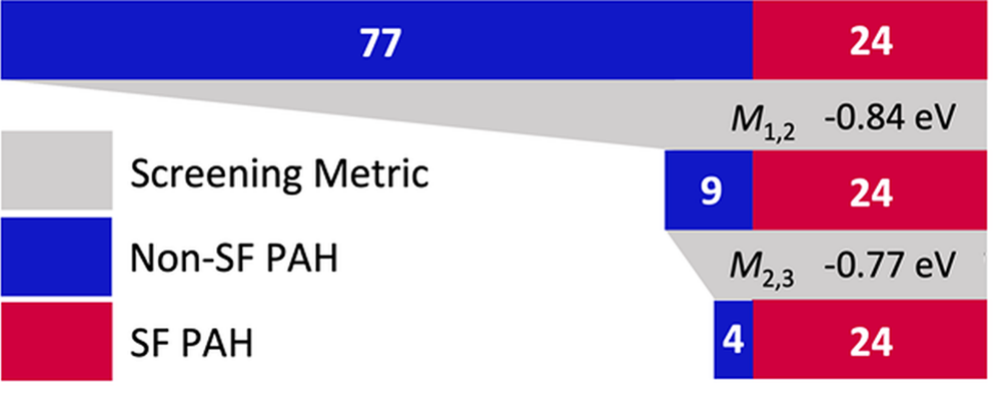
Schematic illustration of the two-stage classifier based on the *M*_1,2_ and *M*_2,3_ SISSO models. The classification thresholds used for each model are shown along with the number of true (red) and false (blue) SF materials out of the PAH101 set that passed each stage of filtering. Reproduced with permission from ref (117). Copyright 2022 Springer Nature.

SISSO produced several models with varying degrees of complexity that were able to predict the GW+BSE SF driving force with a root-mean-square error (RMSE) of ∼0.2 eV or less for the training set and the test set. Based on considerations of model accuracy vs computational cost, two of the SISSO models were selected to construct a hierarchical classifier, illustrated in Figure 12. The thresholds for classifying a material as promising or not promising for SF were set to allow for a small number of false positives and no false negatives, in order to avoid losing any potential candidates. The GW+BSE SF driving force of orthorhombic rubrene, −0.62 eV, was taken as the true positive threshold. Of the PAH101 set, 24 materials are within the positive range and the remaining 77 are considered negative for SF. Only materials that pass the two-stage classifier would be further evaluated with GW+BSE.

**Figure 13:**
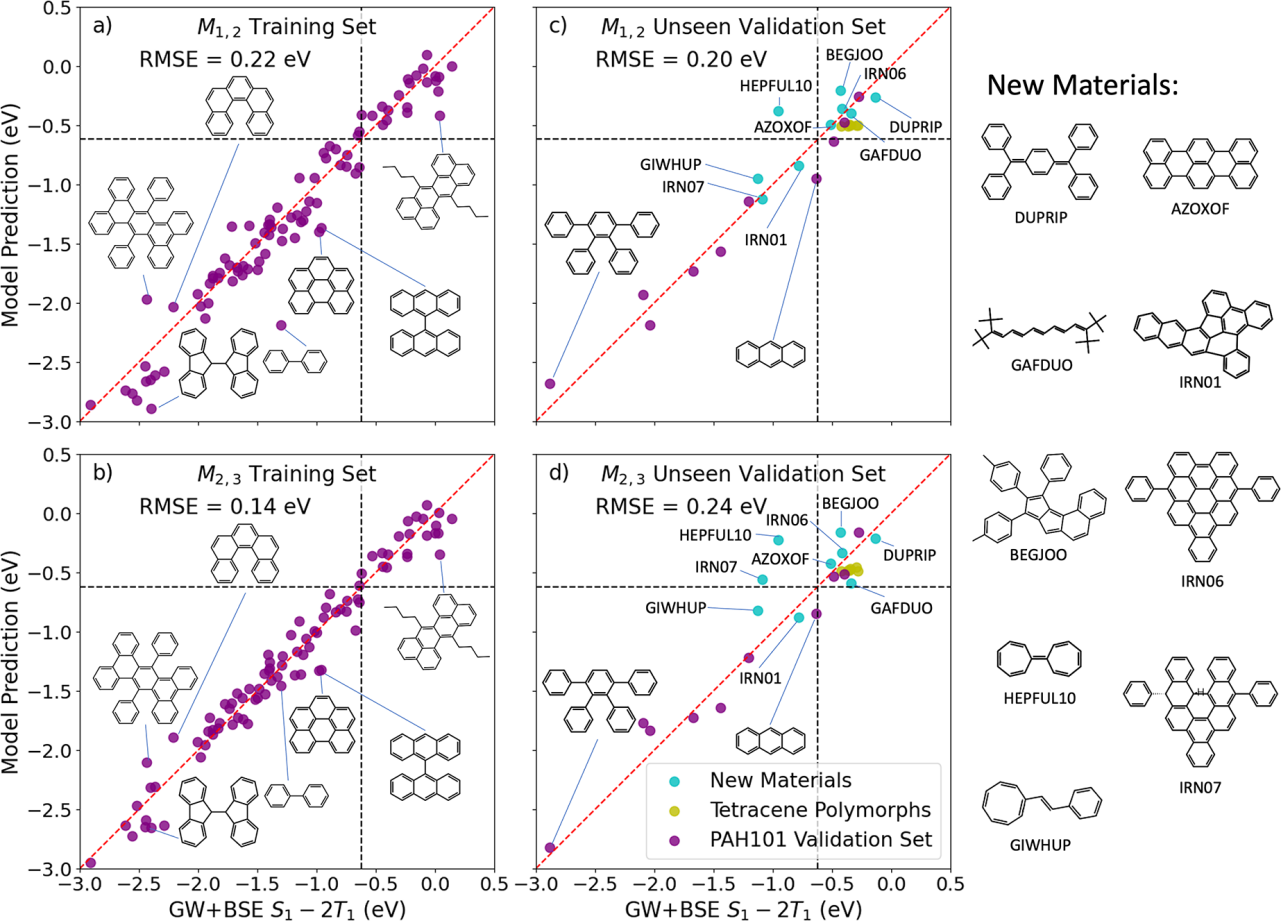
Correlation between the SISSO model predictions and the GW+BSE reference values: (a) *M*_1,2_ for the PAH101 training set, (b) *M*_2,3_ for the PAH101 training set, (c) *M*_1,2_ for an unseen validation set, and (d) *M*_2,3_ for an unseen validation set. Results for the PAH101 set are from ref (117). Chemical diagrams of the new materials tested here and selected materials from PAH101 are also shown.

The low-cost model *M*_1,2_ was selected for the first stage of screening:
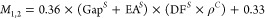
16where Gap^*S*^ is the single molecule DFT HOMO–LUMO gap, EA^*S*^ is the DFT electron affinity, which corresponds to the total energy difference between the neutral molecule and anion, DF^*S*^ is a DFT estimate for the SF driving force with Gap^*S*^ used for *E*_*S*_ and the single molecule DFT triplet formation energy (i.e., the total energy difference between the molecule in the ground state and in the triplet state) used for *E*_*T*_, and ρ^*C*^ is the crystal density. We note that DF^*S*^ is a crude DFT estimate of the single molecule SF driving force, which is not, in and of itself, sufficiently predictive of the crystal’s GW+BSE SF driving force. Evaluating *M*_1,2_ only requires three DFT calculations for a single molecule and the crystal density, which requires no calculations. The *M*_1,2_ model has a training set RMSE of 0.22 eV and a slightly higher test set RMSE of 0.25 eV. Figure 13a shows a parity plot between the SF driving force predicted by *M*_1,2_ and the GW+BSE reference data. The few significant outliers are compounds with different structural/chemical characteristics than most of the PAH101 set, comprising benzene rings connected by single C–C bonds, rather than fused, or long side chains. The classification threshold for *M*_1,2_ was set to −0.84 eV by subtracting the training RMSE of 0.22 eV from the true positive threshold of −0.62 eV. With this threshold all 24 SF candidates in the PAH101 set and 9 other materials passed the first stage of screening. Thus, the low-cost *M*_1,2_ model already eliminated the vast majority of non-SF materials in the data set.

For the second stage of screening we selected the *M*_2,3_ model:
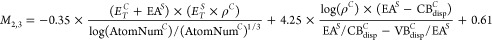
17Most of the features comprising *M*_2,3_ are crystal features, including the crystal density ρ^*C*^, the number of atoms in the unit cell AtomNum^*C*^, the conduction band and valence band dispersion extracted from the DFT band structure,  and , and the crystal triplet formation energy, , which corresponds to the DFT total energy difference between the crystal in the ground state and in the triplet state. The only single molecule features included in *M*_2,3_ are the electron affinity EA^S^ and the triplet formation energy, . The computational cost of *M*_2,3_ is about 20 times higher than that of *M*_1,2_. *M*_2,3_ delivers a significantly improved accuracy with a training set RMSE of 0.15 eV and a test set RMSE of 0.18 eV. Figure 13b shows a parity plot between the SF driving force predicted by *M*_2,3_ and the GW+BSE reference data. Compared to *M*_1,2_, *M*_2,3_ does not produce any significant outliers and performs well even for the systems that are somewhat different than most of the PAH101 set. The classification threshold for *M*_2,3_ was set to −0.77 eV by subtracting the training RMSE of 0.15 eV from the true positive threshold of −0.62 eV. With this threshold most of the false positives from the first stage of screening were eliminated, leaving only 4 false positives. We note that owing to the high computational cost of GW+BSE, each false positive eliminated may save up to a million CPU hours (i.e., the wall clock time it takes to complete the calculation multiplied by the number of CPU cores used), depending on system size.

Panels c and d of Figure 13 show the performance of *M*_1,2_ and *M*_2,3_ for a set of materials not included in the PAH101 training set. These include the original unseen validation set out of PAH101,^117^ the putative polymorphs of tetracene from ref (119), terrylene,^100^ the PAHs from ref (118), 8,9-bis(4-methylphenyl)-10-phenylpentaleno[1,2-*a*]naphthalene (BEGJOO), 3,6-bis(diphenylmethylene)-1,4-cyclohexadiene (DUPRIP), heptafulvalene (HEPFUL10), 3,12-di-*tert*-butyl-2,2,13,13-tetramethyltetradeca-3,5,7,9,11-pentaene (GAFDUO), and (*E*)-1-cyclooctatetraenyl-2-phenylethene (GIWHUP). For the latter five materials, whose structures and CSD reference codes are provided in Figure 13, GW+BSE results are presented here for the first time. A full account is provided in the Supporting Information. We note that some of these new materials are structurally very different from the materials in PAH101, containing, e.g., 7- and 8-membered carbon rings.

Figure 13c shows that *M*_1,2_ performs consistently well for the unseen validation set, including the new materials that were not in the PAH101 set, with an RMSE of 0.20 eV. The only significant outlier is HEPFUL10, whose structure with two 7-membered rings attached by a double C=C bond is very different from the materials in the PAH101 set. With the threshold of −0.84 eV, only HEPFUL10 is misclassified as a promising material for SF (false positive). IRN01, classified as a potential TTA material in ref (118), falls just below the *M*_1,2_ threshold. A drawback of *M*_1,2_ is that because it is largely based on single molecule features, it cannot resolve the differences between the putative polymorphs of tetracene.

Figure 13d shows that the performance of *M*_2,3_ for the new materials is worse than for the PAH101 validation set with an overall RMSE of 0.24 eV. For the nine new materials that were not in the PAH101 validation set, *M*_2,3_ has an RMSE of 0.34 eV, whereas *M*_1,2_ has an RMSE of 0.22 eV. Some of the *M*_2,3_ model predictions deviate significantly from the GW+BSE reference values. The degradation in the model performance may be attributed to overfitting, which is more likely to occur in more complex models, as discussed in ref (117). With the threshold of −0.77 eV, *M*_2,3_ misclassifies HEPFUL10 and IRN07 as promising materials for SF (false positives). With the hierarchical workflow shown in Figure 12*M*_1,2_ would already filter out IRN07, so no improvement at all would be achieved by further screening with *M*_2,3_. Also disappointing is the performance of *M*_2,3_ for the tetracene polymorphs. Despite containing some crystal features, the model predictions vary only slightly across the polymorph set. We note that there were only three polymorphic structures in the original PAH101 training set, which might explain the model’s insensitivity to changes in the crystal structure. To obtain better ML models, more GW+BSE training data needs to be acquired, focusing on increasing the structural and chemical diversity as well as more polymorphic structures.

Of the new materials studied here, three are within the range we consider as promising for SF: BEGJOO, GAFDUO, and DUPRIP. Their GW+BSE SF driving force values are −0.43, −0.34, and −0.136 eV, respectively. BEGJOO and GAFDUO are close to tetracene, and DUPRIP has a higher driving force than pentacene. GAFDUO comprises a long polyene chain, reminiscent of carotenoids, some of which are known to exhibit SF.^36,269−271^ Its optical gap of 3.1 eV and triplet energy above 1.5 eV are probably too high to be of practical usefulness. The triplet energy of DUPRIP, 1.22 eV, could be suitable for pairing with silicon cells. The triplet energy of BEGJOO, 1.07 eV, might be slightly too low but could be paired with other absorbers, as discussed above. Both BEGJOO and DUPRIP do not resemble any known SF materials. This highlights the advantage of computer simulations for identifying potential SF candidates from new chemical families.

## Conclusion

### Summary

The development of SF-augmented photovoltaic devices requires materials that have high SF quantum yields, fast SF rates, large absorption coefficients, appropriate triplet energies to pair with common absorbers, and chemical and photochemical stability under operating conditions. It is hard to find materials that meet all of these stringent demands. Currently, known SF materials are limited to only a few chemical families. In this Review, we have demonstrated how the discovery of new solid-state SF materials can be accelerated by computer simulations employing GW+BSE, the state-of-the-art method for calculating the excited-state properties of crystalline materials with periodic boundary conditions.

GW+BSE can provide information on the band structure, the singlet and triplet excitation energies, the optical absorption spectrum, and the exciton wave function distributions. All of these properties are affected by the crystal packing. Therefore, to rigorously assess the suitability of candidate SF materials, it is not sufficient to consider only the properties of isolated molecules. The primary criterion for assessing how likely a candidate material is to undergo SF is the energetic driving force, *S*_1_ – 2*T*_1_. As a secondary criterion, we have proposed the degree of charge transfer character (%CT) of the singlet exciton wave function, based on the rationale that a high CT character would facilitate the coupling with the triplet exciton states of neighboring molecules. To quantitatively describe the spatial distribution of the two-particle exciton wave function, we developed the double-Bader analysis (DBA) method. We used GW+BSE calculations to evaluate candidate materials, compared to known SF materials, with respect to *S*_1_ – 2*T*_1_ and %CT. For materials deemed as promising, we further considered their triplet energies compared to the band gap of Si and PbSe nanoparticles as representative absorbers for triplet harvesting, as well as whether there is an absorption peak at their optical gap.

We have employed three strategies for the discovery of new SF materials: (i) functionalization of known materials to tune their properties, (ii) finding potential polymorphs with improved crystal packing, and (iii) exploring new classes of materials. All of these strategies have been successful in producing new promising candidate SF materials. The first strategy was exemplified by the exploration of phenylated and pyrene-fused acene derivatives. We have shown that the addition of side groups affects the excitonic properties of molecular crystals by changing the excitation energies of the isolated molecule and changing the crystal packing. After the publication of our predictions, SF was indeed experimentally observed in some pyrene-fused substituted acenes.

The second strategy was exemplified by searching for tetracene polymorphs with improved SF performance by using a property-based genetic algorithm tailored to optimize the stability and SF rate simultaneously. Owing to the high computational cost of GW+BSE, the low-cost dimer model implemented in Simple^258^ was used to evaluate SF rates within the GA, and GW+BSE calculations were performed only for the final candidates. This led to the discovery of a putative polymorph of tetracene with higher *S*_1_ – 2*T*_1_ and %CT than both known crystal structures, which is sufficiently close in energy to the common form to be synthesizable.

The third strategy was exemplified by using GW+BSE for small-scale exploration of rylenes, zethrene derivatives, and a few other PAHs. However, the high computational cost of GW+BSE calculations for molecular crystals is prohibitive for large-scale materials screening. We therefore used the SISSO machine learning algorithm to produce low-cost models that can accurately predict the results of GW+BSE calculations of the SF driving force. To train the models, we generated a first of its kind data set (PAH101) of GW+BSE calculations for 101 PAH molecular crystals with up to 500 atoms in the unit cell. Three additional SF candidates, unrelated to previously known chemical families, were found in the PAH101 set. Here, we tested the performance of the SISSO-generated models for some additional materials not included in the PAH101 set. This revealed three new SF candidates, unrelated to any previously explored chemical family, presented here for the first time (CSD reference codes BEGJOO, GAFDUO, and DUPRIP).

### Outlook

In this Review, we have demonstrated the tremendous potential of computational exploration to discover new chemical families of SF candidates, that would not otherwise be discovered by modification of known compounds or “chemical intuition”, as well as new putative polymorphs. We believe that the future of computational discovery of crystalline SF materials lies in hierarchical workflows combining GW+BSE with low-cost models for preliminary large-scale exploration of chemical compounds and/or crystal structures. As demonstrated here, low-cost models can be either physical models or machine learned models. It can be challenging to improve the accuracy of physical models without significantly increasing their computational cost. Improving the accuracy and transferability of ML models requires acquiring a large amount of training data, which is computationally expensive, and the risk of overfitting may increase with the model complexity. When some of these challenges are overcome, the rate of computational prediction of new SF candidates may exceed the rate of experimental confirmation. At that point, it could be useful to prioritize candidate materials by performing additional detailed simulations beyond GW+BSE to investigate, for example, geometry relaxation in the excited state, entropic effects, interactions with phonons, exciton dynamics, exciton transport, competing processes, and perhaps even photochemical stability. Ultimately, candidate materials have to be tested experimentally to confirm theoretical predictions or, conversely, reveal what improvements are needed. We hope that some of the materials we have proposed will be pursued experimentally. In conclusion, there are exciting prospects for accelerating the development of practical SF-augmented solar cells with the help of computational materials discovery.

## Supporting Information Available

Computational details and results of the GW+BSE calculations for 8,9-bis(4-methylphenyl)-10-phenylpentaleno[1,2-*a*]naphthalene (BEGJOO), 3,6-bis(diphenylmethylene)-1,4-cyclohexadiene (DUPRIP), heptafulvalene (HEPFUL10), 3,12-di-*tert*-butyl-2,2,13,13-tetramethyltetradeca-3,5,7,9,11-pentaene (GAFDUO), and (*E*)-1-cyclooctatetraenyl-2-phenylethene (GIWHUP); primary features and SISSO model predictions for materials not included in the PAH101 set (PDF)
