# Supplementary material for: Computational Discovery of Intermolecular Singlet Fission Materials Using Many-Body Perturbation Theory
Source: J Phys Chem C Nanomater Interfaces. 2024 May 1;128(19):7841–64. doi: 10.1021/acs.jpcc.4c01340 (PMC11103713; doi:10.1021/acs.jpcc.4c01340)
Supplement: Supplementary file 1 — jp4c01340_si_001.pdf [file jp4c01340_si_001.pdf]

Supplementary Information to: Computational Discovery of Intermolecular Singlet Fission Materials Using Many-Body Perturbation Theory

Xiaopeng Wang,[†,‡] Siyu Gao,[¶] Yiqun Luo,[§] Xingyu Liu,[¶] Rithwik Tom,[§] Kaiji Zhao,[¶] Vincent Chang,[¶] and Noa Marom[*,¶,§,‖]

†*School of Foundational Education, University of Health and Rehabilitation Sciences, Qingdao 266113, China.*

‡*Qingdao Institute for Theoretical and Computational Sciences, Institute of Frontier and Interdisciplinary Science, Shandong University, Qingdao, Shandong 266237, P. R. China.*

¶*Department of Materials Science and Engineering, Carnegie Mellon University, Pittsburgh, PA 15213, USA.*

§*Department of Physics, Carnegie Mellon University, Pittsburgh, PA 15213, USA.*

‖*Department of Chemistry, Carnegie Mellon University, Pittsburgh, PA 15213, USA.*

E-mail: nmarom@andrew.cmu.edu

GW+BSE Calculations of Additional Materials

Computational Details

GW+BSE, as implemented in the BerkeleyGW code,[1] was used here to evaluate the excited-state properties of Singlet Fission candidates. To obtain the input wave-functions for *GW*+BSE calculations, mean-field DFT calculations using the PBE functional were performed with the Quantum ESPRESSO code.[2] Coarse k-grid selection based on 20 divided by the lattice constants along a, b and c directions in angstrom. This number was converged based on the GW gap energy difference under 0.1 eV. Thus, a coarse k-grid of 4×2×4, 2×1×2, 2×4×4, 4×4×4, and 2×4×4 was used in the mean field calculations of DUPRIP, BEGJOO, GAFDUO, HEPFUL10, GIWHUP respectively. We used Troullier-Martins norm-conserving pseudo-potentials.[3] The kinetic energy cutoff was set to 50 Ry. The RPA dielectric matrix and the electron self-energy within the *GW* approximation used the coarse grid wave functions as input. 548 unoccupied bands were included. The BSE was solved within the Tamm–Dancoff approximation (TDA). 40 valence bands and 40 conduction bands were included in the BSE calculation. Taking the full dielectric matrix as input to screen the attraction between the electron (e) and hole (h), the e–h interaction kernel was constructed on the coarse k-point grid. To construct the Bethe–Salpeter Hamiltonian, the *GW* quasiparticle energies and e–h interaction kernel calculated with coarse k-point settings were interpolated onto the fine k-point grid of 8×4×8, 4×2×4 and 4×8×8, 8×8×8, and 4×8×8 which are doubles of the coarse grid. The subsequent diagonalization yielded the excitation energies and wave-functions. Absorption spectra were calculated for the SF candidates DUPRIP, BEGJOO, and GAFDUO for light polarized along the three crystal axes and averaged.

Results

Excitation energies

Table S1: GW+BSE singlet and triplet excitation energies of the set of unseen materials, not included in the PAH101 set. The values for terrylene (AZOXOF) are from Ref.,[4] the values for IRN01, IRN06, and IRN07 are from Ref.,[5] and the values for the putative tetracene polymorphs are from Ref.[6]

CSD Code	E_{S1} (eV)	E_{T1} (eV)	$E_{S1} - 2E_{T1}$ (eV)
DUPRIP	2.3	1.22	-0.14
GAFDUO	3.1	1.72	-0.34
BEGJOO	1.71	1.07	-0.43
HEPFUL10	1.89	1.42	-0.96
GIWHUP	3.07	2.1	-1.13
AZOXOF	1.81	1.16	-0.51
IRN01	2.28	1.53	-0.78
IRN06	1.75	1.08	-0.42
IRN07	2.9	1.99	-1.09
TETCEN-P1	2.29	1.33	-0.37
TETCEN-P2	2.29	1.35	-0.42
TETCEN-P3	2.38	1.33	-0.28
TETCEN-P4	2.37	1.36	-0.35
TETCEN-P5	2.44	1.37	-0.29
TETCEN-P6	2.31	1.33	-0.35

Absorption spectra

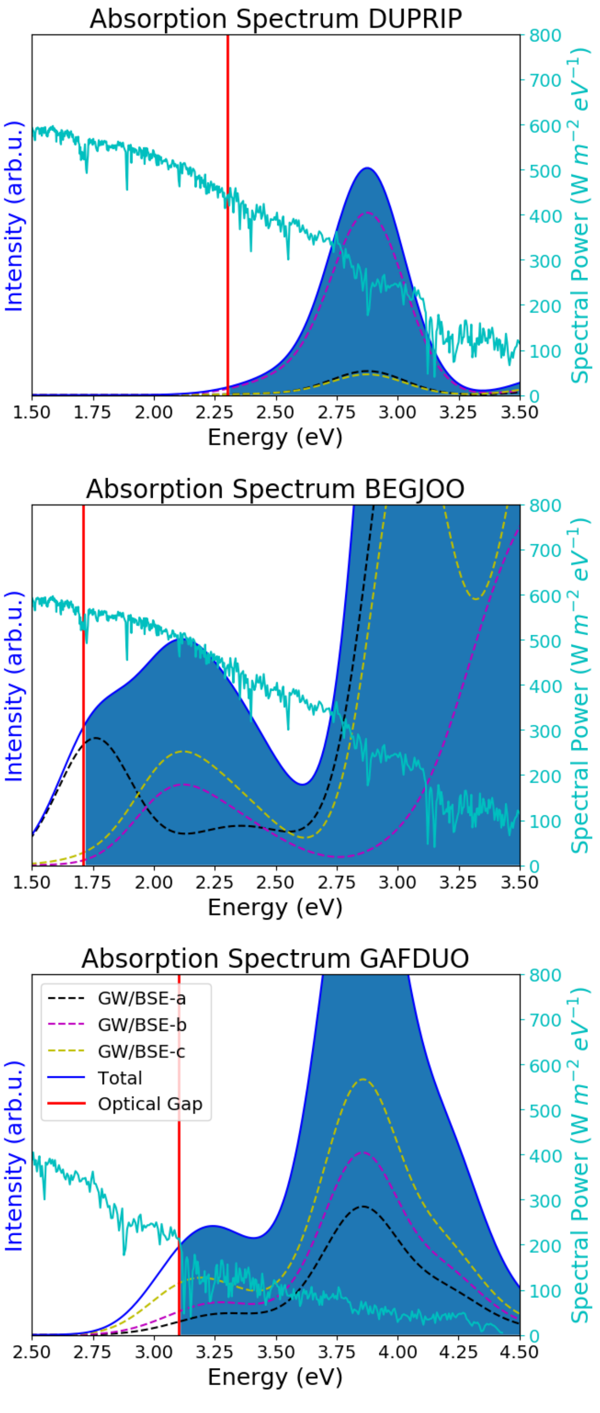

Figure S1: The GW+BSE@PBE absorption spectra of DUPRIP, BEGJOO, and GAFDUO. The red vertical lines indicate the optical gaps. The solar spectrum is also shown.

SISSO Results for New Unseen Materials

Table S2: SISSO $M_{1,2}$ primary features of new unseen materials

*

CSD Code	Gap^S (eV)	EA^S (eV)	E_T^S (eV)	DF^S (eV)	ρ^C ($amu/Å^3$)	$M_{1,2}DF$ (eV)
DUPRIP	1.37	1.47	1.06	-0.75	0.77	-0.26
GAFDUO	1.87	0.92	1.52	-1.17	0.62	-0.4
BEGJOO	0.94	1.83	0.82	-0.7	0.77	-0.21
HEPFUL10	1.5	0.66	1.36	-1.22	0.74	-0.38
GIWHUP	2.16	0.83	1.89	-1.61	0.74	-0.95
AZOXOF	1.26	1.64	1.07	-0.88	0.9	-0.49
IRN01	1.54	1.72	1.35	-1.15	0.87	-0.84
IRN06	1.17	1.76	0.96	-0.76	0.86	-0.36
IRN07	2.07	1.16	1.82	-1.58	0.79	-1.13
TETCEN-P1	1.62	1.17	1.33	-1.03	0.81	-0.51
TETCEN-P2	1.62	1.17	1.33	-1.03	0.81	-0.51
TETCEN-P3	1.63	1.17	1.33	-1.03	0.8	-0.5
TETCEN-P4	1.63	1.17	1.33	-1.03	0.8	-0.5
TETCEN-P5	1.63	1.17	1.33	-1.03	0.8	-0.5
TETCEN-P6	1.63	1.17	1.33	-1.03	0.8	-0.5

Table S3: SISSO $M_{2,3}$ primary features of new unseen materials

CSD Code	$AtomNum$	E_T^C (eV)	VB_{disp} (eV)	CB_{disp} (eV)	$M_{2,3}DF$ (eV)
DUPRIP	56.0	1.06	0.16	0.13	-0.21
GAFDUO	70.0	1.52	0.06	0.31	-0.59
BEGJOO	248.0	0.77	0.05	0.11	-0.16
HEPFUL10	52.0	1.22	0.35	0.2	-0.23
GIWHUP	60.0	1.85	0.21	0.17	-0.82
AZOXOF	184.0	1.02	0.18	0.13	-0.42
IRN01	208.0	1.23	0.07	0.31	-0.88
IRN06	560.0	0.65	0.12	0.05	-0.33
IRN07	148.0	0.87	0.11	0.09	-0.56
TETCEN-P1	60.0	1.21	0.33	0.34	-0.48
TETCEN-P2	60.0	1.22	0.23	0.35	-0.49
TETCEN-P3	60.0	1.24	0.31	0.33	-0.49
TETCEN-P4	60.0	1.22	0.2	0.32	-0.47
TETCEN-P5	60.0	1.27	0.28	0.25	-0.46
TETCEN-P6	60.0	1.21	0.36	0.33	-0.48

References

(1) Deslippe, J.; Samsonidze, G.; Strubbe, D. A.; Jain, M.; Cohen, M. L.; Louie, S. G. BerkeleyGW: A massively parallel computer package for the calculation of the quasiparticle and optical properties of materials and nanostructures. *Comput. Phys. Commun.* **2012**, *183*, 1269–1289.

(2) Giannozzi, P.; Baroni, S.; Bonini, N.; Calandra, M.; Car, R.; Cavazzoni, C.; Ceresoli, D.; Chiarotti, G. L.; Cococcioni, M.; Dabo, I.; others QUANTUM ESPRESSO: a modular and open-source software project for quantum simulations of materials. *J. Phys. Condens. Matter* **2009**, *21*, 395502.

(3) Troullier, N.; Martins, J. L. Efficient pseudopotentials for plane-wave calculations. *Phys. Rev. B* **1991**, *43*, 1993.

(4) Hall, C. L.; Andrusenko, I.; Potticary, J.; Gao, S.; Liu, X.; Schmidt, W.; Marom, N.; Mugnaioli, E.; Gemmi, M.; Hall, S. R. 3D electron diffraction structure determination of terrylene, a promising candidate for intermolecular singlet fission. *ChemPhysChem* **2021**, *22*, 1631–1637.

(5) Andrusenko, I.; Hall, C. L.; Mugnaioli, E.; Potticary, J.; Hall, S. R.; Schmidt, W.; Gao, S.; Zhao, K.; Marom, N.; Gemmi, M. True molecular conformation and structure determination by three-dimensional electron diffraction of PAH by-products potentially useful for electronic applications. *IUCrJ* **2023**, *10*, 131–142.

(6) Tom, R.; Gao, S.; Yang, Y.; Zhao, K.; Bier, I.; Buchanan, E. A.; Zaykov, A.; Havlas, Z.; Michl, J.; Marom, N. Inverse Design of Tetracene Polymorphs with Enhanced Singlet Fission Performance by Property-Based Genetic Algorithm Optimization. *Chemistry of Materials* **2023**, *35*, 1373–1386.
